# Chronic Wound Healing: Research Advances from Pathological Mechanisms to Natural Herbal Active Ingredients and Material Delivery Systems

**DOI:** 10.3390/molecules31061024

**Published:** 2026-03-19

**Authors:** Mengqing Yuan, Yufeng Liu, Xiaoyin Peng, Zhenjun Li, Mingsheng Lei

**Affiliations:** 1 School of Medicine, Jishou University, Jishou 416000, China; 2023700605@stu.jsu.edu.cn (M.Y.); yufengliu2000@163.com (Y.L.); 2 School of Medicine, Hunan Normal University, Changsha 410081, China; 202520264198@hunnu.edu.cn (X.P.); lzjdushenpuaoling@outlook.com (Z.L.)

**Keywords:** chronic wounds, skin healing, dressings, hydrogels, active ingredients

## Abstract

Chronic wound healing is a complex pathological process driven by multiple factors, presenting a significant global healthcare challenge. It not only severely compromises patients’ quality of life but also imposes a substantial socioeconomic burden. In recent years, with deepening insights into the wound microenvironment, composite therapeutic strategies combining natural herbal medicines and their active components with modern biomaterials have offered novel approaches to overcoming refractory wounds caused by diabetic ulcers, vascular lesions, burns, and infections. This paper first outlines the biological foundations of normal wound healing, emphasizing the core mechanisms underlying chronic wound persistence—including persistent inflammatory responses, impaired tissue repair, and cellular dysfunction. Building upon this foundation, the article systematically reviews the existing therapeutic approaches (such as conventional debridement) before focusing on the classification and application of novel biomaterials. It further analyzes the synergistic therapeutic advantages of using materials as delivery systems for natural bioactive compounds. This combined approach enables targeted regulation of the chronic wound microenvironment, synergistically promoting cell proliferation and migration to accelerate healing. Deepening our understanding of the biological mechanisms underlying chronic wounds, coupled with advanced biomaterial technologies, will propel clinical treatment toward more precise and efficient outcomes.

## 1. Introduction

At present, whether in developed or developing countries, chronic non-healing wounds pose a significant socioeconomic burden, negatively impact patients’ quality of life [1,2], and aggravate the burden on the society and family, and we generally classify chronic wounds into five common types, namely venous ulcers [3,4], arterial ulcers [5], diabetic ulcers, traumatic ulcers [6], pressure ulcers, as well as wounds resulting from various clinical conditions and disease complications, such as diabetic ulcers [7], pressure ulcers [8], vascular insufficiency [9], autoimmune diseases [10,11], leprosy [12] and tumors [13]. Improper care of these wounds leads to recurrence and can result in limb amputation and death. The cost of pressure injuries in the U.S. exceeded $26.8 billion in 2016 [14], the total cost of treating stress injuries in the UK is £1.4–2.1 billion per year [15], and pressure injuries bring persistent pain [16,17]. Diabetic foot ulcers affect approximately 18.6 million people worldwide each year and are associated with increased amputation and mortality rates [18]. Overall mortality from diabetic foot ulcers is high, approaching 50% at 5 years [19]. The recovery capacity of the elderly is much slower than that of children and young people, making them more vulnerable to chronic wounds [20]. Chronic wounds have become a focus of research and difficulty, and more effective treatments are needed to heal wounds.

The mechanisms of chronic wound formation are intricate and complex, and we have been very interested in exploring the mechanisms involved in tissue repair and regeneration at the cellular, molecular, and genetic levels. In recent years, the number of studies on herbs and active ingredients extracted from herbs to promote chronic wound repair has been increasing, and significant progress has been made. A variety of novel dressings have been developed for wound healing that have shown significant efficacy in combination with medicines. The purpose of this review is to describe in detail the etiology, mechanism of action, and new therapeutic methods of chronic wound healing, to combine the principles of new materials and active ingredients of herbs to promote wound healing with the mechanisms of chronic wounds, and to study their effects on cytokines and signaling pathways to promote wound repair, to provide references for the treatment of chronic hard-to-heal wounds.

## 2. Stages of Wound Repair

### 2.1. Normal Skin Structure and Function

**Figure 1:**
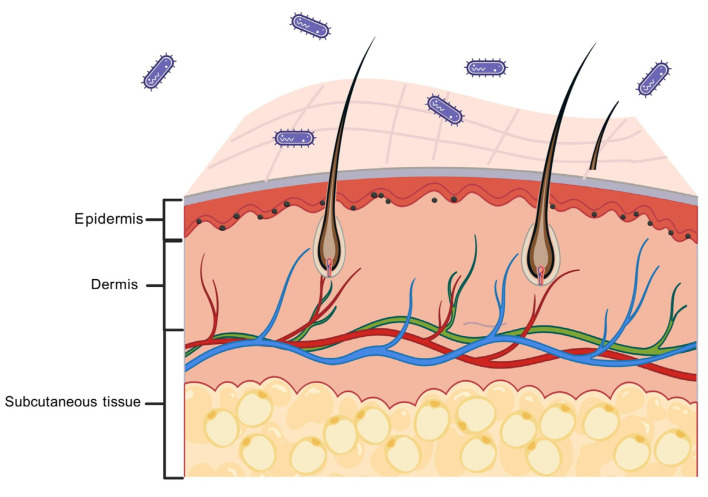
Cross-section of normal skin: epidermis, dermis, and subcutaneous tissue.

In uninjured normal skin (Figure 1), the epidermis is the outermost layer that protects against the complex external environment and prevents the invasion of various harmful substances. The human epidermis consists of the basal layer, the echinocyte layer, the granular layer, the hyaline layer, and the stratum corneum, and also contains sebaceous glands, sweat glands, and hair follicles [21]. The dermis is a layer of tissue below the epidermis, closely connected to the epidermis. The subcutaneous adipose tissue beneath the dermis provides energy for the many types of cells and blood vessels in the dermis [22]. A balanced state of the skin can be achieved by coordinating the various cell and growth factor types within these three layers—epidermis, dermis, and subcutaneous tissue—within normal ranges and at reasonable stages [23].

When skin is injured, the homeostatic balance of the epidermis, dermis, and subcutaneous tissue is disrupted. Trauma causes their separation and loss, and subsequent recovery becomes a lengthy process. During this healing phase, external factors such as infection or pressure on the wound leading to insufficient blood supply, or internal factors like hyperglycemia in diabetes, malnutrition, or aging, can result in chronic wounds that fail to heal despite prolonged treatment, becoming a persistent problem [24].

### 2.2. Physiological Wound Healing

**Figure 2:**
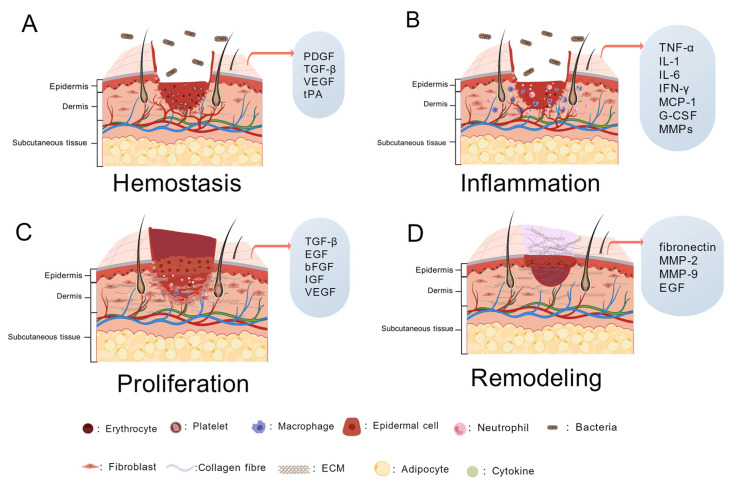
The four stages of normal wound healing. (**A**) Hemostasis. (**B**) Inflammation. (**C**) Proliferation. (**D**) Remodeling.

When the skin is damaged, the body initiates its own healing and repair mechanisms, especially the cells and factors that act on the locally damaged tissue to restore it to its original structure and function. This process can be divided into four phases (Figure 2), mainly including hemostasis, inflammation, proliferation, and remodeling (Table 1). In the normal process of wound repair, the phases can be interconnected and overlapped [23,25,26].

Immediately upon the onset of injury, the body receives signals to restore tissue integrity and homeostasis at the cellular level and smaller molecular levels. In addition, cellular components of the immune system, coagulation cascade responses, and inflammatory pathways are activated. Many types of cells—including immune cells (neutrophils, monocytes, lymphocytes, and mast cells), endothelial cells, keratinocytes, fibroblasts, and the extracellular matrix—are involved in this process [33]. Various growth factors involved in wound healing, such as epidermal growth factor (EGF), fibroblast growth factor (FGF), platelet-derived growth factor (PDGF), transforming growth factor-β (TGF-β), and vascular endothelial growth factor (VEGF), are important players. There are also various other types of cytokines that regulate inflammation and cell growth, and chemokines that direct the directional migration of cells [34].

#### 2.2.1. Hemostasis

Hemostasis is the first stage of wound healing, responding quickly after trauma, accompanied by vasoconstriction and fibrin clot formation, and ultimately the formation of blood clots to exert a hemostatic effect [35]. Vascular endothelial cells release cytokines, and platelets are activated, at which time the endogenous and exogenous coagulation cascade containing biochemical and enzymatic reactions is initiated, and intracellular tissue factor and coagulation factor VII combine to form a complex, ultimately leading to platelet plugs and fibrin plugs, which play a role in hemostasis [36,37].

Platelets have a crucial role in the wound healing process. Their main function is to adhere and activate the formation of hemostatic plugs in the area of bleeding, and platelets contain a variety of signaling molecules and receptors that continue to regulate the healing cascade, but also promote the inflammatory response to guide the subsequent inflammatory stages [38]. Extracellular matrix (ECM) is a three-dimensional structure composed of extracellular macromolecules, formed by the combined action of epidermal and dermal cells, that provides physical support and scaffolding for information transfer to tissues and organs, and structure and growth factors to surrounding cells [39,40,41]. ECM proteins can be categorized as collagen, proteoglycans (PGs), and glycoproteins. Collagen is the predominant structural protein and is classified as fibrillar (collagens I–III, V, and XI) and non-fibrillar, with collagen I making up the interstitial matrix and collagen type IV making up the basement membrane [31]. The hemostatic function of platelets occurs when they come into contact with collagen in the extracellular matrix (ECM), releasing adenosine diphosphate (ADP) to trigger platelet aggregation. Once aggregated, the platelets bind to the collagen network of the ECM, forming a more robust hemostatic scaffold [42].

In the field of regenerative medicine, two platelet concentrates, platelet-rich plasma (PRP) and platelet-rich fibrin (PRF), are used clinically to secrete autologous growth factors that significantly accelerate wound healing [43]. Thrombin is one of the most potent activators of platelets. Thrombin converts fibrinogen to fibrin to promote coagulation, and platelet- and fibrin-rich clots form a healing scaffold and subsequent conversion of soluble fibrinogen to insoluble fibrin polymers [44,45]. Platelets loaded with growth factors and chemokines secreted by other cells and in the plasma adhere to the damaged vascular endothelium and play a hemostatic and reparative role [46].

#### 2.2.2. Inflammatory

The inflammatory process of the wound will have exudate production; exudate composition is very complex, and contains a variety of nutrients and waste products that need to be removed, as well as a variety of cells, inflammatory factors enzymes, and other substances; too much or too little exudate will affect the normal process of wound healing so that the inflammatory period of the wound is prolonged or even not the end of the wound, resulting in the formation of chronic trauma [47]. The fibrin clot formed during hemostasis provides a scaffold for subsequent pro- and anti-inflammatory cells to lodge in the wound. Neutrophils are immediately recruited into the wound area [23]. Neutrophils are among the shorter-lived white blood cells that originate from stem cells in the bone marrow and differentiate and mature into the blood and tissues. Neutrophils are two-fold; a lack of them can cause bacteria to reside on the wound and cause infection, and too much degranulation can lead to an excessive inflammatory response [48]. Neutrophils remove bacteria and dead cells that should not be present in the wound area while releasing reactive oxygen species and cytokines extracellularly to kill extracellular bacteria and recruit leukocytes to the wound area [35].

The persistence of neutrophils in the wound region acts as the clearance of wound foreign bodies and pathogens by the expression of pro-inflammatory factors, proteases, and neutrophil extracellular entrapment networks (NETs) [49]. Pathogens activate platelets via Toll-like receptor (TLR4) while stimulating neutrophils to release NET. Pathogen-associated factors such as bacterial lipopolysaccharide (LPS) stimulate neutrophils, leading to NET release and causing neutrophils to indirectly bind to platelets and activate neutrophil αmβ2 binding to platelet GPIbα to activate the platelets and promote platelet response [37].

Monocytes are recruited immediately after injury and transformed into activated macrophages at the wound site. Macrophages can be categorized into two types, classically activated (M1) and alternatively activated (M2). M1 macrophages are closely associated with pro-inflammatory; in contrast, M2 macrophages have anti-inflammatory, pro-angiogenic functions. M2 macrophages can be divided into four subgroups, M2a, M2b, M2c, and M2d. Of these, M2d involves the transformation of M1-type into M2-type due to the fact that, in the Toll-like receptor (TLR) agonists, it is in the presence of adenosine A2A-stimulated adenosine receptor (A2AR) coactivation results [50]. Type M1 activation is required during the acute inflammatory phase but is also prevalent in chronic wounds, where it expresses CD86 and produces pro-inflammatory factors that assist in the inflammatory response to wounds. Type M2 macrophages express CD206, dectin-1, interleukin-10 (IL-10), and transforming growth factor β (TGF-β), which facilitate the elimination of inflammation in order to allow subsequent angiogenesis and collagen synthesis, promoting proliferation and remodeling. However, trying to differentiate macrophage phenotypes by M1/M2 markers is undesirable, due to the interference of tissue-resident macrophages [27,51].

Healthy skin contains a large number of T cells, including intrinsic γδ T cells and natural killer T (NKT) cells, as well as conventional helper (CD4+) T cells and cytotoxic (CD8+) T cells. T cells are important regulators of inflammatory and reparative responses, producing a number of factors affecting macrophages and fibroblasts, and providing signals to macrophages or fibroblasts. They are produced by the thymus and exported to the periphery, and regulate immunity by suppressing the activation and proliferation of potentially autoreactive T cells present in the normal organism through active regulation. In particular, regulatory T cells (Tregs)—one of the important factors in maintaining the body’s immunoregulatory mechanism—also act through immunomodulation throughout the wound healing process. Among them, tissue-resident Tregs express EGF-like growth factor bi-regulatory proteins that directly promote wound healing and do not pass through the immune perspective, and T cell-related bi-regulatory proteins also induce the production of TGF-β, which promotes wound repair [52]. CD4+ T cells regulate local intrinsic immune activation, in particular monocyte infiltration, and recent evidence suggests that CD4+ T cells can influence the healing and scarring process, especially for repair and remodeling of the heart after myocardial infarction [53,54]. Natural killer T cells (NKT) and neutrophils respond similarly to inflammation by promoting inflammatory responses, but NKT also modulates neutrophils to prevent high-intensity wound inflammation [55,56]. The wound healing process is also co-regulated by CD4+ and CD8+ T-lymphocytes, but they have opposite roles in wound healing, with CD4+ T-lymphocytes promoting healing and CD8+ lymphocytes having a toxic effect on the cells and hindering healing. If both CD4+ and CD8+ T cells are absent, the process of wound healing does not stop [57]. CD4+T participates in the inflammatory response of wounds through its ability to scavenge microorganisms and also control PMNs so that they infiltrate and migrate to the site of infection in response to chemokines, and is also able to produce IL-8, which promotes neutrophil infiltration to the site of inflammation [58].

During the inflammatory phase, immune cells regulate the synthesis, activity, and degradation of the extracellular matrix (ECM). The binding of monocytes to collagen in the ECM promotes the phagocytosis of ECM fragments and other waste products by macrophages [59].

In the later stages of inflammation, macrophages not only phagocytose and digest tissue debris and residual neutrophils but also secrete growth factors and cytokines that promote tissue proliferation and cell migration. The reduction in inflammation and the repair of wound healing are closely linked to a number of macrophage-derived dual-action cytokines, the most representative of which is TGF-β, which directly promotes the wound healing process while inhibiting local inflammation, but in excess inhibits proliferation [27]. L. Steven Beck et al. found that when methylprednisolone was administered to rats or rabbits to impair the healing response, single or multiple topical applications of TGF-β1 reversed the impaired healing secondary to methylprednisolone, and that TGF-β1 acted synergistically with other growth factors to generate granulation tissue and accelerate re-epithelialization [60]. When used in incisions or open wounds, i.e., TGF-β1 can act as an accelerator of wound repair in patients with impaired healing. TGF-β can be secreted by activated macrophages [61]. The main isoforms are TGF-β1, TGF-β2, and TGF-β3, and TGF-β1 is significantly upregulated in acute wound sites while remaining low in chronic wounds, and TGF-β2 and TGF-β3 expression is elevated [62]. The ratio of the three TGF-β isoforms in the same individual is critical for the control of scar formation [63]. The ECM is a three-dimensional structure formed by structural domains composed of proteins that can transmit several complex signals, and ECM fragments in the inflammatory phase activate a variety of immune cells to release pro-inflammatory factors, thereby controlling the propagation and progression of the inflammatory response [64]. Mesenchymal stem cells (MSCs) have immunosuppressive characteristics; they are carried by the bloodstream to the area of injury and inhibit the activation and proliferation of T- and B-lymphocytes, and also release anti-inflammatory factors to assist in the proliferation phase [65].

The action of inflammatory cells and their cytokines released during wound repair contributes to fibrosis and scar tissue formation. The aim of clinical treatment should be to achieve an optimal balance between protective functions. Wound healing becomes chronic if the repair process is stalled for a variety of reasons, such as due to dysfunction of the immune system or a chronic inflammatory state (diabetic foot and stress injuries).

#### 2.2.3. Proliferation

With the immunosuppressive effect of mesenchymal stem cells (MSCs), macrophages gradually shift to M2-type macrophages [66], inflammation gradually subsides, and the proliferative phase begins [28]. The proliferative phase of wound healing generally follows and overlaps with the inflammatory phase and is characterized by the formation of granulation tissue, collagen fibers, and neo-capillaries [67]. Angiogenesis is an essential component of tissue repair, due to the fact that blood vessels provide nutrients and oxygen to the tissues at the wound site and also transport metabolic wastes. Fibroblast growth factor (FGF) promotes the division and proliferation of fibroblasts and also induces angiogenesis and lymphangiogenesis, even affecting the branching pattern of blood vessels [68].

Re-epithelialization is the term used to describe the reappearance of newborn epithelium in skin wounds, lasting from the proliferative phase of the wound to the remodeling phase. Its appearance repairs the barrier function of the skin, which is not possible without the activation of keratin-forming cells and their combination with the ECM to provide nutrients for the reconstruction of the new epithelium [29]. Keratinocytes migrate and proliferate at the wound margin to prolong the newly formed epithelium composed of several layers of cells [69]. The focus of the re-epithelialization process is the keratinocytes, which are the main building blocks of the epidermis, maintaining the integrity of the epidermis in the normal course of events, repairing wounds, and forming new epidermis after injury [70]. Keratinocytes proliferate and produce keratins as they traverse from the basal lamina, like the differentiated lamina; basal lamina keratinocytes express keratins K5 and K14, whereas differentiated keratinocytes express K1 and K10 [70]. The keratinocytes, fibroblasts, and extracellular matrix can interact with each other to produce cytokines such as TGF-α, TGF-β, GMCSF, bFGF, HGF, IL-6, IL-1, etc., to promote wound healing [71]. During wound repair, signals from fibroblasts are received in the form of TGF-β to induce the expression of K5 and K14 in keratinocytes to complete the repair of the basal lamina [29]. If TGF-β1 is not involved in re-epithelialization, keratinocytes hyperproliferate but stimulate epithelial regeneration at low doses of TGF-β1, and inhibit epithelial regeneration at high doses of TGF-β1 [72,73]. TGF-β1 signaling regulates integrins by inducing epidermal keratinocytes, thereby promoting the adhesion and migration of keratinocytes to migratory components of re-epithelialization, i.e., to fibronectin [74,75]. Exosomal PD-L1 promotes wound healing from an immunosuppressive perspective; it inhibits the production of cytokines by CD8+ T cells, which can promote the migration of epidermal cells and fibroblasts, accelerating wound contraction and re-epithelialization [76].

Integrins are heterodimeric receptors in which different α and β subunits can form 24 integrins capable of both recognizing components in the ECM and enabling cell adhesion to the extracellular matrix [77]; they also act as a cell surface receptor for direct transduction of ECM signaling [78]. IL-1 induction in keratinocytes stimulates fibroblasts to synthesize cytokines such as FGF7 and IL-6, which in turn stimulate the proliferation of keratinocytes in a dual paracrine manner, forming a co-culture system. In addition, in response to pro-inflammatory factors or growth factors, fibroblasts are transformed into myofibroblasts, appearing in the granulation tissue [79].

Matrix metalloproteinases (MMPs) are a large family of calcium- and zinc ion-dependent endopeptidases [80], a class of secreted enzymes with broad-spectrum protein hydrolytic activity, which regulates inflammation and tissue repair, and can degrade almost all protein components in the extracellular matrix (ECM), which also contributes to the remodeling of the ECM [81]. Blood clotting during hemostasis provides the wound with ECM proteins to assist in cell migration; keratinocytes require ECM to assist in re-epithelialization, and fibroblasts pull on the ECM, causing the wound to contract and form a scar [82]. Cells in the resting state exhibit low levels of MMPs, but when cytokines and growth factors stimulate the cells during infection or tissue remodeling, MMP levels increase rapidly.

During collagen degradation, collagen types I and III are first cleaved by MMP-1 and MMP-8, and collagen type IV is degraded by MMP-9 [83]. Collagen fibers provide tensile strength to the ECM, provide elasticity to the tissue, contribute to scar formation, and provide support for angiogenesis. They are now used as drug delivery scaffolds or incorporated into dressings for wound repair [84]. The proteoglycan family of proteoglycans, acetyl heparin sulfate proteoglycans (HSPGs), are reservoirs of growth factors, and heparin can bind to many growth factors (e.g., FGFs, VEGF, etc.) [77,85].

Glycoproteins such as laminin, elastin, fibronectin, and platelet reactive proteins have multiple functions. In addition to their role in ECM assembly, they participate in ECM–cell interactions by acting as ligands for cell surface receptors such as integrin [81]. Interestingly, periosteal proteins were significantly upregulated during ECM remodeling in skin injury [86].

The transmission of cellular signals during the healing process relies not only on the 3D structure of the ECM but also requires the assistance of the pericellular matrix (PCM). The PCM is a region formed around cells, with its location and composition varying depending on cell type. Acting as a scaffold, it facilitates cell communication and nutrient metabolism through components like integrins and proteoglycans. It contains numerous molecules capable of recognizing growth factors and transporting them to the cell surface, thereby controlling the timing of their binding to receptors [87,88].

At the end of the proliferative phase, myofibroblasts converted from fibroblasts interact with the ECM, and the wound shrinks [23], but the predominant force for wound contraction is the production of collagen fibers by the fibroblasts, which use traction to arrange the granulation tissue [89].

#### 2.2.4. Remodeling

The proliferative phase of granulation tissue coincides with the early stages of wound remodeling for wound maturation, which represents the onset of wound maturation, with clinical manifestations including wound contraction, reduction in erythema and hardness, and increase in the strength and organization of the epidermis [90]. There is a balance between wound integrity and degree of scarring, and collagen remodeling during the transition from granulation tissue to scarring is dependent on collagen degradation controlled by MMPs, with different subtypes of collagen synthesized and catabolized at lower rates [26]. Collagen I is synthesized while collagen III is cleaved, and the granulation tissue, composed of collagen III, stops growing [23]. Most of the cells actively involved in wound healing (neutrophils, macrophages, myofibroblasts, etc.) undergo apoptosis at this stage and are expelled from the wound, leaving a small number of cells, and reduced collagen and other ECM proteins [33]. Gradients of chemokines influence the movement of fibroblasts in the scar; fibroblasts need to maintain their own movement, and if regulated by ECM composition and direction, can lead to uneven development of the scar matrix [91]. As the scar matures, the capillaries within the wound degenerate and become less dense, and are occupied by dense collagen fibers [90]. Due to the complexity of the skin, there is a need for more beneficial research methods and solutions, whether traditional or advanced, to fully improve skin wound healing [92]. Therapies based on growth factors such as VEGF, PDGF, bFGF, HGF, etc., have already been put into the real world of medical treatment and may become a completely basic treatment in the near future [93].

### 2.3. Chronic Wound Healing

The staging and overlapping of wound healing are regular processes, and when wound recovery fails to pass through this sequential process, healing of the skin tissues is delayed, eventually leading to chronic wounds. Common features are exudation, recurrent infections, difficulty in re-epithelialization, decreased angiogenesis, and hyperplasia [94]. When normal repair responses are impaired, there are two primary outcomes: an ulcerative skin defect and excessive scar formation. The overexpression of large numbers of neutrophils, pro-inflammatory macrophages, and pro-inflammatory factors (e.g., TNF-α and IL-1β) contributes to the persistence of chronic wounds. In addition, the situation is further complicated by persistent inflammation and delayed healing of bacterial biofilms, which perpetuates the inflammatory period [94].

The vascular system is the infrastructure for achieving immunomodulatory function and adequate damage repair. When the vasculature in diabetic foot ulcers (DFUs) is not functioning properly, vascular endothelial growth factor (VEGF) aids in angiogenesis to promote wound healing [95], and oxygen intervenes in the VEGF of the skin damage [96]. Ischemia and sustained pressure are the main etiologies of pressure ulcers, where sustained pressure and shear from self or medical devices can lead to prolonged ischemia and obstruction of venous capillary flow in the skin, resulting in tissue necrosis in severe cases [97].

Inflammatory factors play an important role in wound healing, and deficiencies or excessive amounts can cause delays in healing. Studies have shown that interleukin-6 (IL-6)-deficient mice are more susceptible to bacterial infections and have a diminished inflammatory response to injury, with delayed re-epithelialization [98]. The pro-inflammatory macrophage phenotype does not transition to an anti-inflammatory macrophage phenotype; pro-inflammatory factors are present in abundance, the inflammatory phase is prolonged indefinitely, and pro-inflammatory factors inhibit the function of anti-inflammatory factor [99].

Excessive and prolonged TGF-β1 at the wound site is detrimental to wound healing and is present throughout almost the entire wound healing process, affecting not only inflammatory cells but also controlling the re-epithelialization of keratinocytes and fibroblasts [100]. Abnormal reductions in TGF-β receptors have been found in chronic diabetic ulcers, and even in undamaged diabetic skin, TGF-β1 is lower than in normal skin. Thus, in diabetic ulcers, the lack of TGF-β1 may lead to a severe disruption of the normal healing process [101].

The steady-state equilibrium of ECM production, turnover, and degradation in chronic wounds is disrupted, triggering abnormalities in a series of proteins, including growth factors, matrix metalloproteinases, collagen, and glycoproteins [102]. One of the characteristics of chronic wounds is increased matrix metalloproteinase activity and impaired production and activation of MMPs. Different MMPs have different effects on wound healing, and wound healing can be impaired if broad-spectrum MMP inhibitors are used [80]. MMPs disrupt and break down the integrity of the ECM and the growth factors contained therein. In the absence of tissue inhibitors of metalloproteinases (TIMPs), MMPs are unregulated, and cellular growth is reduced in chronic wounds involved in multiple causes. Chronic wound macrophages release a variety of matrix metalloproteinases (MMPs), particularly MMP-2 and MMP-9, to prevent the proliferative phase of healing [101]. The proteolytic destruction of ECM not only prevents the wound from moving forward into the proliferative phase but also attracts more inflammatory cells and amplifies the inflammatory cycle [29]. Dysregulation of the balance presented by MMPs and their inhibitors (TIMPs) can occur with the development of hyperplastic scarring (excessive accumulation of the ECM) or the formation of chronic wounds (excessive degradation of the ECM) [28].

Integrins manage intracellular and extracellular growth factor (TGF-β, VEGF, FGF) signaling, and modulation of collagen-binding integrin activity may also be a way to improve chronic wound healing through fibrosis [30]. During the re-epithelialization phase, αvβ6 integrins preserve the function of keratinocytes from the impeding effect of hyperglycemia on wound healing in diabetic wounds [103].

## 3. Factors Affecting Wound Healing

**Figure 3:**
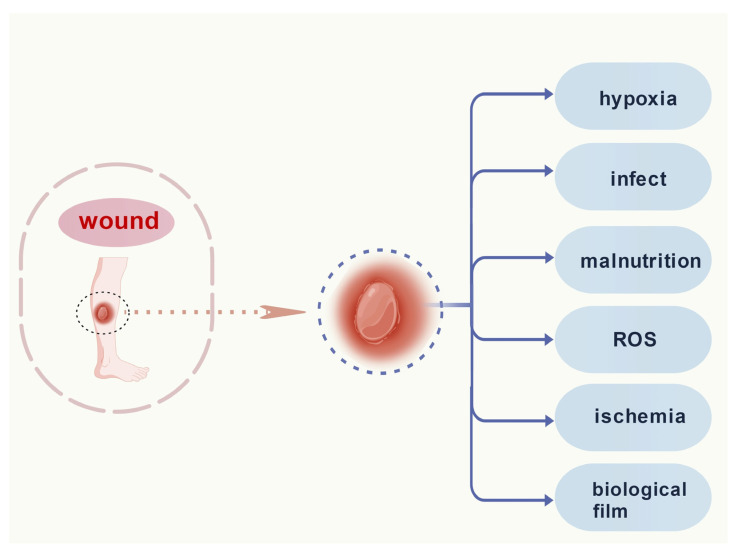
Factors affecting wound healing. Infection and biofilm formation, hypoxia and excessive ROS production, inadequate blood supply, and malnutrition are detrimental to wound healing.

Common factors contributing to chronic wounds are recurrent bacterial infections, reduced angiogenesis, impaired tissue epithelialization, and ROS overload, which is also associated with permanent ischemia and potentially higher oxidative stress (Figure 3). Clinically, infected wounds present with symptoms of redness, swelling, heat, and pain, sometimes occurring with the presence of purulent exudate in the wound or increased wound drainage, which spreads to form a more severe condition in severe cases [104]. The inflammatory is manifested as the presence of neutrophils and pro-inflammatory macrophages in the wound. Decreased neutrophil apoptosis and increased levels of neutrophil-associated proteases, especially elastases and MMPs that degrade the ECM, as well as the chemokine IL-8, which acts through cytotoxicity on neutrophils to achieve its modulation of the inflammatory response, have all been implicated in the formation of chronic cutaneous wounds. The pro-inflammatory M1 macrophages reside in the wound and do not polarize into pro-healing anti-inflammatory M2 macrophages, which are associated with chronic skin wound formation. The prolonged presence of inflammatory cells results in an indefinitely prolonged inflammatory phase in the chronic wound healing process, which is enhanced by mast cell and CD8+ T cell activity, and elevated levels of other inflammatory T cell subsets such as Th1, Th17, and Th22. All these pathological processes together promote inflammation, tissue fibrosis, and new vascular structures [49]. The wound healing process is influenced by both local and systemic factors. On the one hand, local factors directly affect wounds, including radiation, hypothermia, infection, tissue oxygen tension, and pain. On the other hand, systemic factors are related to the degree of the body’s overall health status, which affects an individual’s ability to heal wounds.

### 3.1. Infections and Biofilms

The presence of bacteria in wounds is a serious problem; the distribution of bacteria in wounds is heterogeneous, and the degree to which wound healing is impeded is increasing with the continued emergence of multidrug-resistant Pseudomonas aeruginosa and Staphylococcus aureus. Bacteria exist in complex adherent communities called bacterial biofilms, which are complexes of bacteria encapsulated in an extracellular matrix composed of hydrated polymers and debris. Various major biomolecules, such as proteins and polysaccharides, present in biofilms are capable of upregulating the levels of pro-inflammatory cytokines, ROS, and MMPs, while decreasing the levels of oxygen content, TIMPs, and growth factors [105].

Infection in chronic wounds of the skin is thought to be associated with biofilms. The persistence of biofilms affects both the inflammatory and reparative phases of the wound, producing excessive pro-inflammatory cytokines and preventing angiogenesis and collagen fiber production [106]. Biofilm adheres firmly to the tissues surrounding the wound, and the difficulty in addressing biofilm is that it is not only difficult to eradicate at the surface, but it can lurk along the perivascular perimeter and below the wound surface. Microorganisms are not present singly in wounds, but rather a multiplicity of microorganisms coexist. Superficial easy-to-heal wounds contain more staphylococci, mainly Staphylococcus aureus, while deeper hard-to-heal wounds contain more anaerobes and Gram-negative amoebae [107]. Biofilms are not significantly effective in the face of antibiotic treatment, and the difficulty in removing Pseudomonas aeruginosa using both antibiotics and fungicides is due to the fact that it grows as a biofilm in the deeper parts of the wound. Optimized debridement can be used as an adjunctive modality to make the biofilm more sensitive to external agents, as well as to target treatment by using PCR to identify biofilm-forming bacteria [108,109].

### 3.2. Hypoxia-Ischemia and Excess Reactive Oxygen Species (ROS)

Oxygen is necessary for every step in the healing process. Wound healing requires oxygen to interact with numerous cytokines, and the processes of cell proliferation, angiogenesis, and collagen synthesis need to be supported by adequate oxygen, with important considerations being oxygen supply and tissue oxygen consumption. Oxygenation of traumatized tissue depends on both the oxygen supply capacity of the traumatized tissue, the density of the traumatized tissue and its surrounding capillaries, and the rate of oxygen consumption of the constituent cells in the traumatized tissue. Post-traumatic vasoconstriction and extremely active inflammatory cells consume oxygen, cause hypoxia, and produce ROS [110]. It has been shown that under acute hypoxic conditions, keratinocytes and fibroblasts have stronger collagen synthesis, which translates into faster activation and migration of cells that help initiate wound healing [111]. ROS can disrupt the function of many normal cells by destroying nucleic acids, oxidizing proteins, etc. Low levels of ROS have a defensive effect against microorganisms and also promote cell migration and angiogenesis; however, oxidative stress caused by excess ROS leads to the oxidation of lipids, proteins, and nucleic acids, and, in particular, in the persistent inflammatory response to chronic wounds a highly pro-oxidative microenvironment exists in which ROS are be stored in excess, which can lead to cell death and even systemic injury in severe cases. Measures to remove excess ROS from wounds to reduce oxidative stress can shorten the time required for chronic wounds to heal. Oxygenation or the use of oxygenated dressings has been shown to be beneficial for chronic wound healing and can provide future options for chronic wound healing [112,113]. Treatment of chronic wounds must ensure that the wound microenvironment is maintained in a state of homeostasis, keeping ROS at normal levels, and thus breaking down persistent inflammation to allow for a normal transition to a proliferative phase [114].

### 3.3. Ischemia

Angiogenesis and blood infiltration are essential for chronic wounds, providing nutrients and media to the nascent granulation while removing metabolic waste products. Lower doses of VEGF applied to ischemic wounds detected higher vascular density compared to the high-dose group and the control group [115]. Inadequate angiogenesis results in delayed wound closure, reduced epithelialization, and defective granulation tissue formation. Angiopoietin COMP-Ang1 protein, a form of bone oligomeric matrix protein, accelerates angiogenesis and lymphangiogenesis in ear wounds and increases epidermal and dermal regeneration and blood flow in tail skin wounds in diabetic mice [116]. Granulation tissue formation in ischemic wounds of the rabbit ear decreased from 63% to 39%, and infection increased from 2% to 20%, whereas these negative changes were absent in congestive wounds [117]. The use of extracorporeal shock wave therapy (ESWT) in diabetic rats with STZ-induced dorsal skin defects significantly increased blood perfusion to the traumatic area during the early healing phase, and the expression of VEGF and eNOS in the traumatic margin area was significantly increased, allowing for the formation of neovascularization, a decrease in localized inflammatory response, and a decrease in leukocyte infiltration [118].

Inadequate blood supply also affects the levels of growth factors such as TGF-β and K-FGF, limiting the activation of growth factors to the point where they do not function to promote angiogenesis and collagen synthesis, which is a sequential process [119]. Fluid supply and proper circulation are essential for wound healing.

### 3.4. Poor Nutritional Status

Patients with chronic wounds are affected by their body’s ability to absorb and their quality of life. While nutritional deficiencies may be less evident in acute wound recovery due to poor self-absorption or lack of good social conditions for obtaining adequate nutrients, recovery from chronic wounds can be profoundly affected by nutrient availability. Adequate nutrition is required during the inflammatory, proliferative, and remodeling phases for cell proliferation, angiogenesis, collagen synthesis, etc., in the wound [120]; as a result, malnutrition prevents early wound healing, slows down wound healing, increases the risk of infection, and prolongs the time it takes to reach a better level of strength during the healing process [121]. However, nutrient supply above the normal range has no additional benefit for wound healing [122]. Supplementation of immune nutritional formulas such as arginine, glutamine, and vitamins can improve wound healing efficiency in terms of immune function. Glutamine supplementation reduces infection, arginine increases collagen synthesis, and vitamin supplementation, such as vitamin C, plays a key role in collagen formation and stabilization. Micronutrients such as zinc and selenium act as antioxidants and are also beneficial for wound healing [123]. The cost of wound care and repair should not be overlooked, and nutritional availability should be emphasized in wound repair.

## 4. Treatment

### 4.1. Traditional Methods

#### 4.1.1. Debridement

Various traditional methods have been tried in the field of wound healing to avoid infection and pain and to promote tissue repair. Debridement is a fundamental and powerful tool for wound healing, removing senescent and necrotic cells, removing blood clots and foreign bodies present in the wound, and avoiding bacterial colonization [124]. Surgical debridement is more effective than treatment with antibiotics alone in a relatively short course of time [125], especially in chronic wounds, where some debris can be a breeding ground for bacterial colonization, and easily identifiable inactivated and necrotic tissues can be excised with a scalpel until fresh, blood-perfused tissue remains [126]. Severe infections in pressure ulcer wounds result in a high rate of lethality due to sepsis, so focus on assessing the risk of debridement [127]. A variety of new and appropriate debridement modalities have emerged to date. The waterjet debridement system is a system in which a jet of high-pressure sterile saline is used to clean the wound, and then the saline mixed with debridement waste is pumped out. Waterjet debridement in burns, lower extremity venous ulcers, and pressure ulcers significantly reduces debridement time, minimizes repeat debridement, and has potential cost-saving benefits [128]. Solutions commonly used to clean wounds can be saline or topical antiseptics including hydrogen peroxide solution, Dakin’s solution, povidone-iodine, bleach boric acid solution, alcohol, acetic acid, and chlorhexidine, which kill and remove bacteria, but most of these solutions are toxic and should not be used for long periods of time, and there is no evidence of healing-promoting effects [129].

#### 4.1.2. Gauze

Traditional wound dressings usually consist of low-cost sterile pads and gauze; however, traditional dry gauze wound dressings may cause wounds to be susceptible to bacterial infections and tend to adhere to the wound, and fresh granulation tissue is destroyed during dressing changes, which can also cause further damage and lead to a less-than-optimal wound healing process [130].

### 4.2. Moisturizing Dressings

There is a growing clinical need for wound dressings and an increasing demand for smart wound dressings. The tremendous advances in the field of biomaterials and the deeper understanding of the wound healing process have given rise to many new therapeutic approaches and strategies. Natural or synthetic biomaterials are widely used in biomedical applications to support, enhance, or replace damaged tissues or biological functions. The delivery of biological agents such as scaffolds (scaffolds), hydrogels, or nanoparticles via biomaterials has become prominent in wound care approaches. Artificial dermis based on human fibroblasts and their secretory products can also play an effective role in chronic wounds, especially diabetic ulcers, and in the United States, artificial dermis has been approved as a class III medical device for the treatment of DFUs in stage III [131].

The overall moisturizing dressings available on the market can be divided into five components (Table 2), hydrogels, foams, hydrocolloids, films, and alginates, which play a key role in moisturizing wounds through their water vapor transmission rate (WVTR). Compared to dry wound dressings, the moist wound environment created by the new moisturizing dressings is rich in growth factors that promote autolytic debridement, maintain electrical gradients, activate collagen synthesis, increase angiogenesis, and reduce scarring [132,133,134]. The American College of Physicians (ACP) clinical practice guidelines state that the use of hydrocolloid or foam dressings is equally effective in accelerating wound healing in patients with pressure ulcers and both are superior to gauze dressings, but in a 2013 Cochrane systematic review by Sohan et al. it was reported that the difference between foam dressings for diabetic foot ulcers and conventional treatments was not statistically significant [135].

Wound dressings are the key to exudate management. Wound exudate contains high levels of inflammatory cytokines and chemokines, which are hotbeds for bacterial growth, and dressings protect the localized wound site from further trauma by controlling the level of wound exudate, as well as providing hydration and absorbing excess exudate to provide the most conducive environment for successful healing [136]. Materials used to make dressings should be well biocompatible. In addition to having a direct positive impact on the wound with their own good materials, these materials can also act as local delivery systems to carry useful drugs and replenish cells with their regenerative capacity for chronic wounds [113]. Depending on the type of chronic wound, an appropriate dressing may be used to create a temporary barrier to microorganisms while maintaining moisture within the wound area.

**Table 2:** Description, advantages, disadvantages, and commercially available brands of moisture-retaining dressings.

Dressing	Description	Advantages	Disadvantages	Examples
Hydrogels	Crosslinked hydrophilic polymers with three-dimensional networks [137,138]	High water content; excellent biocompatibility; facilitates autolytic debridement [139]	Poor mechanical properties; low viscosity	Purilon^®^ Gel (Coloplast, DK); Intrasite Gel (Smith & Nephew, UK)
Foams	A porous foam structure primarily composed of polyurethane [140]	Strongly absorbs wound exudate to maintain the wound microenvironment; highly flexible, conforms to the shape of the wound [141,142]	Adhesion to dry wounds with minimal exudate may cause secondary injury	Biatain Silicone (Coloplast, DK); Aquacel™ Ag Foam (ConvaTec, UK); Mepilex^®^ (Mölnlycke Health Care, SE)
Hydrocolloids	The primary component is carboxymethyl cellulose, composed of polymeric hydrogel and other viscous materials [143]	Maintain a moist wound environment to promote granulation tissue formation; high patient comfort [144]	Limited absorbency makes it unsuitable for wounds with heavy exudate	DuoDERM^®^ (ConvaTec, UK); Comfeel^®^ Plus Transparent (Coloplast, DK)
Films	A transparent polymer film coated with an adhesive, typically made of polyurethane [145]	Excellent breathability; isolates bacteria to prevent infection; facilitates observation of wound condition [146]	Poor absorption capacity leads to fluid retention; low elasticity	Tegaderm™ (3M Health Care, Minnesota, USA); Opsite Flexigrid (Smith & Nephew, UK); Mepore^®^ Film (Mölnlycke Health Care, SE)
Alginates	Natural polysaccharides extracted from brown algae [147,148]	Easily gelatinizes; excellent biodegradability; high moisture absorption; hemostatic function [148]	Not suitable for dry wounds or wounds with persistent bleeding; when changing dressings, be mindful of residual dressing fibers	Aquacel™ Extra™ (ConvaTec, UK); Biatain^®^ Alginate (Coloplast, DK)

#### 4.2.1. Hydrogels

Hydrogels are hydrophilic polymers with three-dimensional networks that are structurally and functionally similar to natural ECM. The 3D structure gives hydrogels excellent biocompatibility and protects biologically active molecules from being altered [137,138]. A variety of natural polymer hydrogels and synthetic polymer hydrogels have been developed. Natural polymer hydrogels are widely used in regenerative medicine due to their original inherent biodegradability and cell adhesion properties, and they exhibit a variety of advantages in biomedical applications. Compared with synthetic polymer hydrogels, ECMs have more similar physical and chemical properties to natural biopolymer hydrogels and usually exhibit good cell viability and cell differentiation [139]. Most commonly used in recent hydrogel research are polysaccharides such as chitosan, alginate, hyaluronic acid, and protein-based hydrogels such as collagen and gelatin. Bio-responsive hydrogels are also able to be used in applications such as drug delivery. Hydrogel materials are modified to contain small biomolecules that selectively bind biomolecules, or drug molecules are chemically incorporated into the hydrogel to use the hydrogel as a vehicle for drug delivery [149]. When hydrogels are used as drug carriers, the design is mainly focused on the two properties of injectability and modulation of parameters controlling drug release. Drug delivery from hydrogels is mainly focused on controlling the release of small molecule drugs more accurately, which is affected by the interactions between the carrier and the drug, which can be composed of specific and non-specific interactions such as hydrogen bonding, Schiff base bonding, van der Waals forces, ionic interactions, and hydrophobic interactions [150].

Biomaterials used for hydrogels (e.g., hyaluronic acid, fibronectin, chitosan, gelatin, etc.) [26] are capable of serving as carriers for delivering biological agents to treat chronic wounds through a variety of mechanisms, and successful results have been achieved. Chitosan (CS), one of the most widely used hydrogel materials, is an alkaline hydrolyzed derivative of chitosan with good biocompatibility and low crystallinity but low mechanical properties. Chemical modification of chitosan maintains both the basic skeleton of chitosan and its original properties [151]. Lu et al. prepared a dual-network (DN) hydrogel, using chitosan as the first network and polyacrylamide formed the second network. This dual-network structure has stronger mechanical properties and solubility, which can effectively load inflammation-reducing factors, intelligently regulate wound inflammation, and promote neovascularization [152]. The use of gelatin methacrylate (GelMA), a bifunctionalized gelatin-based Rhodococcus haematobium HEA hydrogel, produces astaxanthin-containing (Astaxanthin (AST)) red hue Rhodococcus haematobium RHEA (RHEA) with multiple ROS-scavenging enzyme activities and antioxidant capacity in bacterial-infected wounds in diabetic mice under photo exposure, using photothermal sterilization, promotion of M2 macrophage polarization to control inflammation, and enhancement of cell proliferation and migration to promote neovascularization [153]. In a mouse model of diabetic infected wounds, polyethylene glycol (PEG)-derived conjugated polyimidazolium salts (PIM) and N-acetylcysteine (NAC) formed a bifunctional hydrogel crosslinked network, PPN, in which the polyimidazolium cation possessed potent antimicrobial ability, and the N-acetylcysteine (NAC) showed excellent antioxidant properties. If alginate is used instead of polyethylene glycol for crosslinking the assembled hydrogel, it is more suitable for deep wounds [154].

In the combination of hydrogels and natural plant active ingredients, okra has potent antioxidant properties that significantly reduce intracellular ROS production, and okra extract-derived hydrogel dressings increase cell migration, angiogenesis, and re-epithelialization in chronic wound areas [155]. The injectable GAAS/Pec@Sr hydrogel, composed of pectin (Pec) extracted from apple peel combined with strontium ions (Sr^2+^) and glycerol ammonium salt (GAAS), is suitable for irregular wounds. This represents a newly developed hydrogel [156]. Commonly used hydrogel dressings include Purilon^®^ Gel (Coloplast) and Intrasite Gel (Smith & Nephew).

Hydrogel-based biomaterials are advanced dressing materials due to their excellent growth factor embedding, controlled release, and functionality. Various studies in recent years have taken advantage of the collective multiple crosslinking of dynamic covalent bonding, metal–catechol chelation, and hydrogen bonding to increase the bioadhesive properties and develop multifunctional hydrogel wound dressings.

#### 4.2.2. Foams

In recent years, foams have received a lot of attention in the treatment of wound dressings. Foam dressings are polymers, usually made of soft polyurethane (PU) and polymethylsiloxane [140]. A variety of PU foams have been used as promising wound dressing materials. The mechanical properties of the foam dressing are the main reason for the functionality of the foam dressing. The porous structure of the foam component contains air, and the void gradient determines its water absorption and swelling characteristics, which absorb the degree and rate of exudate absorption by the dressing. Flexibility determines their friction properties, adhesion, and durability [141,142]. Foam permeability is critical to the flow of wound exudate, especially in composite layers of foam, and influences wound healing through the absorption, transfer, and evaporation of exudate [142]. These physical properties create a moist, incubator-like microenvironment for the wound, which is able to absorb the wound exudate moderately and keep it in a balanced state, keeping the wound surface moist without over-absorption of exudate leading to adhesion between the dressing and the wound, or under-absorption leading to leakage of too much exudate, minimizing the production of scarring. Compared with other foam dressings (32~1000 μm), Medifoam^®^ N has a more uniform and smaller pore size (25~75 μm) than other dressings, which has excellent exudate absorption and retention ability, and Medifoam^®^ N-treated SD rats showed excellent angiogenesis and collagen deposition [157]. Compared to polyurethane, polyurethane urea-based foam (PUUF) is a new rapid hemostatic and mild wound dressing with hydrogen bonding interactions providing better hydrophilic and mechanical properties. It has excellent hydrophilicity, clotting effect, and low toxicity, and enhances re-epithelialization [158]. Polyurethane (pDADMAC-PU) foam wound dressing coated with a highly antimicrobial, high-density quaternary amine salt (poly(diallyldimethylammonium chloride)) showed inhibition of biofilm formation of three pathogens (Staphylococcus aureus, Pseudomonas aeruginosa, and Acinetobacter baumannii) and of biofilm formation of each of the pathogens, in a Quantification of Uptake (CFU) assay [159]. Modification of polyurethane foam with different concentrations of kaolin resulted in a significant increase in hemostatic capacity without affecting the already significant absorption capacity of PU foam [160]. Foam dressings have a preventive effect on pressure ulcers, and the application of multilayered soft silicone foam dressings to prevent sacral and heel pressure ulcers is of clinical significance [161]. Porcine ischemic skin wounds treated by Patil and colleagues using 7 ethylene glycol (EG7) forming polythioketone urethane foam (PTK-UR) variants had higher hydrophilicity, better ROS responsiveness and clearance, lower catabolism of inflammatory factors and pro-inflammatory immune cells, and higher re-epithelialization and ECM when compared to wounds treated with another dressing, NovoSorb BTM production [162].

Clinical outcomes in wound care result not only from the foam properties of the dressing itself but also from the complexity of the dressing design, which has a profound effect on dressing performance. Alginate can be involved in foam formation, based on which Namviriyachote et al. developed polyurethane foam dressings (A6-1Ag-AS) containing silver and asiaticoside, which stimulates collagen synthesis and reduces oxidative stress in wounds and activates fibroblast proliferation, and silver (Ag), which has antimicrobial properties, and so developed a novel material that had a good wound healing effect in a porcine model [163]. Potato-derived asiaticoside (AS) loaded onto polyurethane foam dressings (PUC) is softer than starch (CS) and collagen-derived proteins (Ge). Conveniently, the foam dressing can be loaded with various drugs for wound healing, such as replacing metal ions or other medications like centella asiatica glycosides, enabling greater flexibility in clinical treatments for patients with different conditions [164]. Biatain Silicone (Coloplast), Aquacel™ Ag Foam (Conva-Tec), and Mepilex^®^ (Mölnlycke Health Care) are well-established brands of foam dressings.

#### 4.2.3. Hydrocolloids

Hydrocolloids absorb wound exudate liquid and can form hydrogels under certain conditions. They have the properties of a hydrogel and have been developed for many types of wound healing by applying them to carriers such as foams and films [143]. Hydrocolloids are most used in chronic wounds like lower extremity ulcers, pressure sores, and burns, and also play a role in the management of acute and surgical wounds [165]. Although the use of hydrocolloids generates a corresponding increase in healthcare costs, it reduces treatment time by minimizing the number of dressing changes, improves cure rates, and reduces patient suffering compared to gauze [144]. The hydrocolloid dressing containing filipin nanoparticles (SFNHD) enhanced the physical properties and efficacy of the hydrocolloid dressing compared to gauze and commercially available Neoderm^®^ regenerative repair patches in South Korea, reducing the size of the burn area and increasing the density of collagen fibers in a rat burn model, and immunohistochemistry staining showed an increase in proliferating cell nuclear antigen (PCNA) expression only in the SFNHD dressing when compared to the other groups. (PCNA) expression was increased, indicating burn wound dermal regeneration [166]. Zulfakar et al. developed an alginate-based bilayer hydrocolloid membrane based on the principle of combining the upper layer of the infiltrating drug (ibuprofen) with a simple lower layer, and the MVTR of the bilayer is lower than that of a monolayer, whereupon water molecules diffuse for a longer diffusion time to achieve the effect of carrying the drug and controlling control–release of the drug, which is suitable for use in low septicity wounds [167]. Moringa leaf (MOL) extracts of varying concentrations contain several major active compounds, such as vicine-2, rosmarinic acid, and chlorogenic acid. This natural herbal extract film dressing, formed by combining alginate, glycerol, and pectin, achieved sustained-release effects on wound models in type 2 diabetic rats. Notably, the low-concentration group (0.5% MOL film) reduced TNF-α and monocyte chemotactic protein-1 (MCP-1), factors involved in early inflammatory responses. It also increased VEGF levels during re-epithelialization, demonstrating excellent anti-inflammatory and pro-angiogenic effects [168]. DuoDERM^®^ (ConvaTec) and Comfeel^®^ Plus Transparent (Coloplast) are versatile, easy-to-use hydrocolloid dressings. The ability of hydrocolloids to improve materials that can be obtained by combining various substances is a promising method of wound treatment.

#### 4.2.4. Films

Film dressings are translucent transparent polymer films (usually made of polyurethane) coated with an adhesive [145]. They maintain moisture by reducing water permeability while allowing gas exchange. Natural, non-toxic chitin films are also similar to polyurethane-based film dressings [146]. Film dressings of different material compositions show different needs in the wound healing phase [169]. In chronic wounds, multilayer (LBL) films have been developed for a variety of functions around pH, temperature, and the ability to load drugs [170]. In contrast to the traditional occlusive gauze dressing group, the transparent film dressing can be clinically more effective in reducing surgical site infections during surgery than traditional closed gauze [171].

Zhao et al. prepared a three-layer (LBL) film with good self-healing ability based on dynamic borate bonding, loading KPV and EGF at the bottom and middle layers, with glucose-responsive and water-triggered peelable properties, while regulating inflammation and remodeling phases, which facilitates the healing of diabetic wounds [170]. Li et al. prepared a chitosan-based composite film (CS/CuS/Gent/PCA) containing anthocyanin, copper sulfide, and gentamicin, in which purslane anthocyanin (PCA) is highly sensitive to the pH environment and has bacterial detection ability, and CuS nanoparticles possess a good photothermal effect, and this composite film then provided patients with a timely alert to achieve bacterial killing and promote diabetic wound healing [172]. Films containing thyme oil exhibit significant inhibitory effects against both Gram-positive and Gram-negative bacteria. Furthermore, the antioxidant properties of carvacrol in thyme oil enable the films to be used for antibacterial and antioxidant applications in wound healing [173]. Interestingly, applying a film material over the wound after using herbal medicine is also a viable approach. For instance, Reza Ranjbar et al. applied aloe vera gel to back wounds infected with Staphylococcus aureus in rats, then covered the area with a film. Microbiological examination revealed that this combined approach demonstrated stronger antimicrobial effects than either aloe vera gel or the film alone. It also resulted in higher hydroxyproline levels and smaller wound areas, indicating that re-epithelialization progressed more closely to that of normal wounds [174]. Products from brands such as Tegaderm™ (3M Health Care), Opsite Flexigrid (Smith & Nephew), and Mepore^®^ Film (Mölnlycke Health Care) have been well established in clinical use.

The development of ideal film materials as wound dressings has great potential in the field of medicine.

#### 4.2.5. Alginates

The ability of alginate (ALG) to form gels is the main reason why it is mainly used in soft tissue engineering and wound healing [147]; alginate gels are typically nanoporous gels with high absorption capacity for use in severely exuding deep wounds, and they are also capable of delivering a variety of low molecular weight medications for use in the field of wound healing [148]; one or more bioactive factors promoting wound healing can be incorporated into the alginate dressings so that effective concentrations can be maintained. Alginate can be ionically crosslinked by the addition of divalent cations to an aqueous solution. The chemical nature of alginate and the relatively mild crosslinking conditions allow for a gelation process that allows for the incorporation of a wide range of bioactive substances, including proteins, cells, and DNA oligonucleotides, into the alginate matrix, while retaining intact bioactivity [175]. Alginate can be directly isolated from algae. It serves as both a natural active ingredient and a material for dressings, and can also function as a component in the development of new materials. It possesses antioxidant properties [176]. Nanoscale alginate dressings formed by calcium crosslinking and freeze-drying can be used as delivery platforms for the topical treatment of diabetic wounds hosting polarized macrophages, termed “cellular bandages,” and the overall number of polarized macrophages, particularly M2c macrophages, is increased in dressings-treated diabetic wounds of mice [177]. Alginate is a promising biodegradable polymer that shows great utility and potential as a biomaterial in many biomedical applications; products such as Aquacel™ Extra™ (ConvaTec) and Biatain^®^ Alginate (Coloplast) offer significant advantages. The modification of alginate or its use as a carrier can be useful in wound healing.

### 4.3. Delivery Systems of Biologicals

With the advancement of bioengineering technology, materials can serve as scaffold structures, anti-infective therapeutic agents, and drug delivery carriers, and this technology is applied in the care of chronic wounds. Multiple crosslinked hydrogels with zinc oxide (ZnO), named (CaGOD/ZnO), showed excellent blood compatibility in vitro, and in vivo experiments indicated that the hydrogels rapidly closed rat liver hemorrhagic wounds, and also adhered to the ventricular wall to prevent hemorrhage in rat ventricular perforation models. Bacterial survival of CaGOD/ZnO was very low in a whole skin defect model using methicillin-resistant Staphylococcus aureus (MRSA)-infected rats [178]. Injectable hydrogels (CNC-S) containing cellulose sulfate nanocrystals are able to serve as scaffolds to load vascular endothelial growth factor (VEGF), thereby inducing significant angiogenesis. The sulfate moiety of the hydrogel plays an important role in biocompatibility and controlling the rate of VEGF release [179]. By loading chitosan nanoparticles (FOE-CHNPs) containing blue cordyceps extract (FOE) into calcium alginate hydrogels, significant collagen deposition could be seen in a diabetic rat wound model, and gene expression analysis upregulated VEGF, TGF-β, and b-FGF to promote diabetic wound healing [180]. Desferrioxamine (DFO) was loaded as gelatin microspheres (DFO@G) into chitosan-containing borate ester bonds, forming a hydrogel with self-healing and injectable properties; the borate ester bond reacts with glucose and ROS to reduce hyperglycemia and oxidative stress, and gelatin microspheres can react with matrix metalloproteinase-9 (MMP-9) to slow the release of the drug in them, which can upregulate the expression of hypoxia-inducible factor-1 (HIF-1α) and VEGF expression, accelerating angiogenesis, collagen deposition and rapid wound closure [181]. Micron- and nanoscale particles are also commonly used for drug loading and controlled drug release [182]. In topical drug delivery systems, nanoparticles allow the continuous delivery of substances to localized wounds. Combining materials with nanoparticles has become a new technology in the field of wound healing. For example, metal (Ag, Zn) nanoparticle-coated dressings exhibit antimicrobial activity and play a crucial role in the wound healing process [183]. Delivery systems for biologics have been developed for use in the fields of biotechnology, pharmaceutical research, and smart medicine, and have tremendous growth energy.

### 4.4. The Active Components of Herbal Medicine and Their Combination with Materials

**Figure 4:**
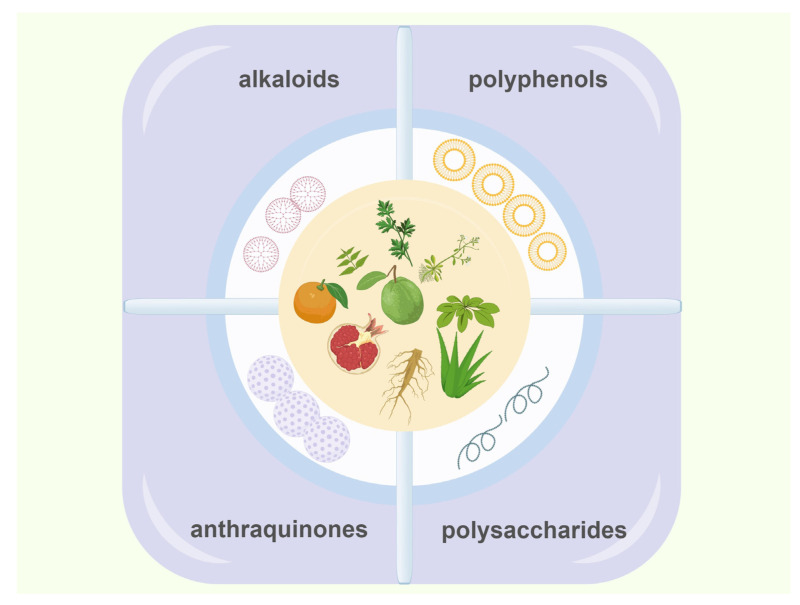
Classification of natural plant bioactive compounds: alkaloids, polyphenols, anthraquinones, and polysaccharides.

The widespread application of natural herbs compounds represents a future trend. It was not until the last century that molecular biology methods and techniques were gradually developed, enabling recognition of the role of growth factors in wound healing. By regulating endogenous growth factors, binding to receptors, and activating downstream signaling pathways, these compounds demonstrate effective therapeutic potential in the treatment of skin ulcerative diseases (Figure 4).

#### 4.4.1. Alkaloids

Coptis chinensis (CC) is a recognized traditional herbal medicine widely used in food and pharmaceutical applications. Berberine (BBR), also known as berberine, is a quaternary isoquinoline alkaloid isolated from the Coptis root, which inhibits LPS-induced macrophage inflammation by blocking the NF-κB, MAPK, and AKT signaling pathways, and it is the main active ingredient of Coptis root’s antimicrobial activity [184]. Berberine has good scavenging activity against hydroxyl radicals (•OH), indicating antioxidant activity [185]. BBR activates TrxR1 and inhibits downstream JNK signaling, thereby inhibiting oxidative stress and apoptosis and promoting cell proliferation. It downregulates matrix metalloproteinase MMP-9 to enhance extracellular matrix (ECM) synthesis and accelerate wound healing [186]. Combining berberine with suitable dressings can enable novel techniques for wound healing. Samadian et al. used electrostatic spinning to prepare berberine-containing cellulose acetate/gelatin (CA/Gel) nanofiber dressings for use as DFU dressings, and in vitro experiments investigated the antimicrobial activity in diabetic rats [187]. Hu et al. prepared a biocomposite hemostatic film with the addition of berberine; the film composition contains alginate, chitosan, and collagen, berberine and calcium chloride act as crosslinking agents, and their combination increases the mechanical properties and hemostatic ability of the material. The combination of berberine and calcium chloride as crosslinking agents increases the mechanical properties and hemostatic capacity of the material [188].

#### 4.4.2. Polyphenols

Polyphenols are natural compounds commonly found in plants that exhibit potent antibacterial and antioxidant properties. Flavonoids constitute a significant proportion of these compounds and have garnered considerable attention and popularity in the pharmaceutical and food industries [189]. In the treatment of chronic wounds, polyphenols can replace commonly used broad-spectrum antibiotics to reduce oxidative stress at the wound site and protect against infection risks while simultaneously mitigating inflammation to facilitate wound healing [190,191].

The primary component in tea leaves, tea polyphenols (TPPs), possesses the ability to promote wound healing at every stage. During the inflammatory phase, it reduces levels of pro-inflammatory cytokines such as IL-1β, IL-6, and TNF-α, and lowers the myeloperoxidase (MPO) content primarily found in neutrophils. In the proliferative phase, it specifically increases VEGF-A levels to accelerate capillary formation and stimulates collagen synthesis for faster new tissue formation [192]. Among these, green tea polyphenols (TPs) demonstrate significant efficacy in wound healing, particularly for diabetic wounds [193]. A hydrogel (TPN@H) combining polyvinyl alcohol and alginate loaded with nano-green tea polyphenols can target and inhibit the PI3K/AKT signaling pathway in diabetic SD rat wounds, thereby suppressing NF-κB pathway expression and reducing inflammation. It also lowers levels of SMA protein, strongly associated with scar formation, preventing excessive scar hyperplasia [194].

Guava leaves (*P. guajava*) contain high concentrations of diverse polyphenols that are safe and non-toxic to humans. The K Bilal team demonstrated through total antioxidant capacity (TAC) and ferric reducing antioxidant power (FRAP) assays. In a rat epidermal excision wound model, the extract (PGLE) reduced hydrogen peroxide-induced oxidative stress and increased antioxidant levels such as catalase (CAT) [195], accelerating wound re-epithelialization.

Curcumin (CNP), a polyphenolic compound, is incorporated via nanotechnology into a hydrogel with hyaluronic acid and chitosan (OHA-CMC), forming an OHA-CMC/CNP/EGF composite hydrogel by loading the herbal active ingredient curcumin (CNP) and EGF. Following the Schiff base reaction, the hydrogel exhibits significant antibacterial properties and stops bleeding. Curcumin was rapidly and sustainedly released to reduce inflammation and oxidative stress in the early stages of wound healing, whereas the slower and sustained release of EGF supported proliferation and ECM remodeling in the later stages. This was confirmed in a diabetic mouse model [196]. Curcumin was loaded into a filipin (SF)-based hydrogel and accompanied by controlled and sustained release, producing higher granulation tissue thickness, collagen deposition, upregulation of vascular endothelial growth factor (VEGF), and a reduced inflammatory response in a model of total skin defects [197].

Three polyphenols—tannic acid (TA), oligomeric proanthocyanidins (OPC), and (−)-epigallocatechin gallate (EGCG)—were incorporated into a composite system. Copper ion Cu^2+^ crosslinked nanoparticles (CuNPs) were combined with a mixture of water-soluble carboxymethyl gellan gum (CMCS) and phenylboronic acid (PBA) via boronic ester bonds to construct a hydrogel-crosslinked polyphenol nanoparticle (CuNPs) matrix containing polyphenols and copper ions. The hydrogel leveraged its physical properties to achieve hemostasis on mouse wounds, while the three polyphenols and Cu^2+^ ions exerted antioxidant and antibacterial effects. This induced polarization of more M2 macrophages, shortening the inflammatory response and promoting collagen synthesis during re-epithelialization [198]. Tannic acid (TA) can even crosslink borate hydrogels to exhibit enhanced broad-spectrum antibacterial activity [199], indicating the significant potential of polyphenols in chronic wound treatment.

#### 4.4.3. Anthraquinones

Rhubarb has a long history as a medicinal herb, with documented effects including diuretic, detoxifying, and heat-dispersing properties. Its primary active components, including emodin, belong to the anthraquinone class and have been found to possess targeted mechanisms against cancer and multi-organ fibrosis [200]. Rhubarb charcoal processed from rhubarb, crosslinked with fibrin sponge material (RCS/SF), demonstrated excellent hemostatic capacity on diabetic mouse wounds. The crosslinked structure’s enhanced physical properties, which resist detachment, further amplify therapeutic efficacy. Among factors positively correlated with angiogenesis—FGF, VEGF, and D31—the rhubarb charcoal–protein sponge group yielded the most favorable data [201]. Further investigation revealed that this composite material modulates AMPK signaling pathways to regulate glucose and lipid metabolism in diabetic mice, demonstrating significant potential for concurrent internal and external diabetes treatment [202]. Emodin, a major anthraquinone compound in rhubarb extract, exhibits potent free radical scavenging activity. When formulated as nano-emodin (N-EMO), it demonstrates efficacy in ultrasonic therapy against bacterial biofilms formed during burn wound infections, inhibiting biofilm growth and eliminating bacteria. Emodin inhibits fibroblast proliferation by suppressing the PI3K/Akt signaling pathway, preventing excessive scar tissue proliferation caused by the overactivation of fibroblasts during the remodeling phase of wound healing in mouse scar models [203]. This effect was further confirmed in rat pulmonary fibrosis models, where it inhibited fibrosis induced by fibroblast migration and proliferation [204].

**Figure 5:**
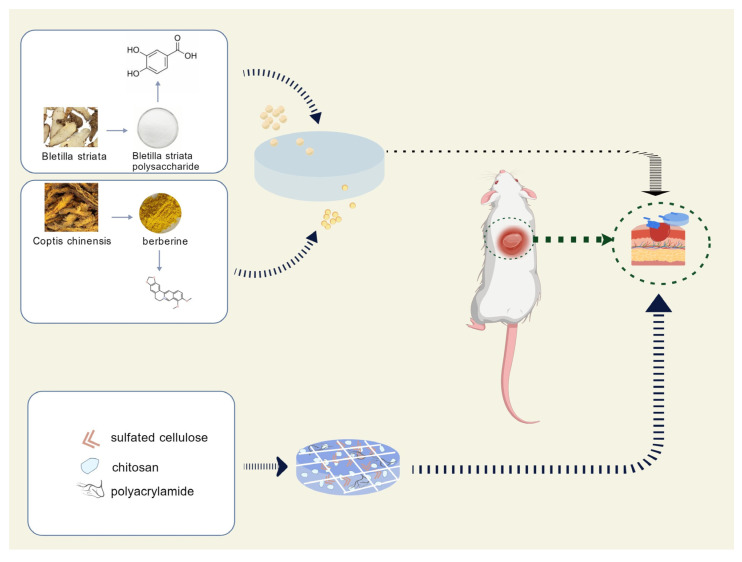
Application of herbal active ingredients combined with materials.

Angelica dahurica extract (AE), Rheum officinale extract (RE), and ethanolic extract of Rheum officinale angelica dahurica (Rheum officinale extract (ARE)) showed significant inhibitory effects against Staphylococcus aureus, suggesting the antibacterial and anti-inflammatory effects of the herbal extracts. More CD31 and VEGF were detected in the ARE group, which effectively promoted angiogenesis, and compared with the presence of more collagen fibers and more myofibroblasts in other groups, accelerated excisional wound healing in rats (Figure 5) [205,206].

#### 4.4.4. Polysaccharides

Polysaccharides, as naturally sourced compounds, are widely utilized in wound healing therapies due to their excellent biocompatibility and degradability. Particularly, their direct loading onto hydrogel materials—where they can undergo covalent and ionic crosslinking with the matrix—has become a prominent research focus [207]. Among these, polysaccharides extracted from herbal medicines exhibit distinct antibacterial and antioxidant advantages, making them a key area of herbal research [208].

Bletilla hyacinthina, a commonly used herb for wound repair, has an excellent ability to stop bleeding and promote angiogenesis [209]. Bletilla striata polysaccharide (BSP) is a high molecular water-soluble polysaccharide obtained by extraction and isolation from the tubers of Bletilla striata of the Orchidaceae family, which has shown good performance as a promising natural biomaterial. BSP can control the expression of pro-inflammatory factors at an appropriate level to reduce inflammatory responses in wounds, and it can disrupt the bacteriostatic function of the cytoplasmic membrane of bacterial cells (e.g., ATCC, MRSA) [209,210]. The surface has a loose filamentary network structure, which allows the preparation of fibers with mechanical properties, and the mechanical strength depends on the preparation method. The fibers have hydroxyl radical scavenging ability and higher reducing sugar content, thus exerting antioxidant ability [211]. Hydrogels with self-healing properties based on oxidized betulinic polysaccharide (BSP) and cationic gelatin (BG-gel) rapidly stop hemorrhage and generate a higher density of neovascularization during subsequent healing in a mouse severed-tail and rat liver incision model [212]. BSP sponges created with cryotechnology have a porous structure, exhibit good adsorption and degradation properties, and have excellent hemostatic capacity [213]. BSP reduced serum levels of inflammatory mediators TNF-α, IL-1β, and IL-6 to varying degrees in a carrageenan-induced inflammatory model of foot swelling in mice, significantly decreased the NO concentration in the serum and liver, and increased superoxide dismutase (SOD) activity, suggesting good anti-inflammatory and antioxidant capacity [214].

The extraction of herbal components and the combination of active ingredients with novel materials applied to chronic wound healing have produced good efficacy. However, the effects of some herbs are not yet completely clear and in-depth, and still need to be further explored so as to fully utilize the efficient role of herbs in promoting wound healing.

## 5. Conclusions

Chronic wound healing remains a challenge in the medical field and we need to find new treatments. On the surface, wound healing is accompanied by a number of changes that are visible to the naked eye, and behind these visible changes are a series of complex processes involving various cells, growth factors, cytokines, and components of the ECM [99]. Acute wounds and chronic wounds are widely present in the development of healthcare, such as mechanical trauma, surgical wounds, and complications in chronically ill patients. Chronic wounds are formed due to nutritional deficiencies, infections, age, and other factors, and the problems faced by patients are not only expensive to treat but also suffer from prolonged physical pain. It is imperative to discover efficient, convenient, and inexpensive treatment methods. A good understanding of the types and proportions of cells involved in normal and chronic wounds, as well as the timing and targeted function of each cell, is the most important background for healing wounds.

Novel materials, natural or synthetic, have shown great ability in chronic wound management, relying on their physical and chemical properties to manage wound exudate and insulate against outside microorganisms to prevent infection, and even acting as scaffolds for loading medications for enhanced therapeutic effects [113]. Natural herbs and their active components also boast a long history and extensive applications. By reducing the persistence of inflammatory factors such as IL-1, IL-6, IFN-γ, and TNF-α, increasing levels of anti-inflammatory factors like IL-4, IL-6, and IL-10, promoting the application of growth factors including EGF, FGF, PDGF, and TGF, and decreasing reactive oxygen species (ROS) production, the therapeutic properties of herbal active ingredients have been explored. This ultimately achieves anti-inflammatory, antioxidant, and regenerative effects. The application of growth factors such as platelet-derived growth factor (PDGF) and transforming growth factor (TGF), coupled with the reduction in reactive oxygen species (ROS) production, achieves anti-inflammatory, antioxidant, and regenerative effects [28]. This treatment has demonstrated remarkable efficacy in skin trauma applications. By integrating novel materials with herbal formulations, it holds great promise for benefiting patients suffering from chronic wound healing disorders caused by various factors. This approach alleviates patient suffering and reduces the burden on families, positioning it as a highly promising clinical treatment pathway in the field of chronic wound care.

## Figures and Tables

**Table 1:** Stages of wound healing and the events.

Phase	Events
Hemostasis [23,24,25]	Blood vessels constrict
	Platelets aggregate to form a clot
	The fibrin network stabilizes the clot
	Minimal cellular activity, mostly platelets and fibrin mesh
Inflammation [27,28]	Blood vessels dilate
	Neutrophils and macrophages invade the wound site
	The area becomes red and swollen due to the increased blood flow and immune cell activity
Proliferation [29,30]	Fibroblasts are actively producing collagen
	New blood vessels form (angiogenesis)
	Keratinocytes migrate to cover the wound
	Granulation tissue, a red and bumpy tissue, starts forming
Remodeling [31,32]	Collagen fibers realign
	Fibroblasts decrease in number
	Scar tissue forms and matures
	The wound contracts and becomes less red as the blood vessels decrease

## Data Availability

No new data were created or analyzed in this study. Data sharing is not applicable to this article.

## References

[1] Klein T.M., Andrees V., Kirsten N., Protz K., Augustin M., Blome C. (2021). Social Participation of People with Chronic Wounds: A Systematic Review. Int. Wound J..

[2] Tan M.L.L., Chin J.S., Madden L., Becker D.L. (2022). Challenges Faced in Developing an Ideal Chronic Wound Model. Expert Opin. Drug Discov..

[3] Krizanova O., Penesova A., Hokynkova A., Pokorna A., Samadian A., Babula P. (2024). Chronic Venous Insufficiency and Venous Leg Ulcers: Aetiology, on the Pathophysiology-Based Treatment. Int. Wound J..

[4] Kelechi T.J., Johnson J.J., Yates S. (2015). Chronic Venous Disease and Venous Leg Ulcers: An Evidence-Based Update. J. Vasc. Nurs..

[5] Broderick C., Pagnamenta F., Forster R. (2020). Dressings and Topical Agents for Arterial Leg Ulcers. Cochrane Database Syst. Rev..

[6] Gilligan G., Panico R., Di Tada C., Piemonte E., Brunotto M. (2020). Clinical and Immunohistochemical Epithelial Profile of Non-Healing Chronic Traumatic Ulcers. Med. Oral Patol. Oral Cir. Bucal.

[7] Burgess J.L., Wyant W.A., Abujamra B.A., Kirsner R.S., Jozic I. (2021). Diabetic Wound-Healing Science. Medicina.

[8] Carter M.J., DaVanzo J., Haught R., Nusgart M., Cartwright D., Fife C.E. (2023). Chronic Wound Prevalence and the Associated Cost of Treatment in Medicare Beneficiaries: Changes between 2014 and 2019. J. Med. Econ..

[9] Skórka M., Bazaliński D., Więch P., Kłęk S., Kozieł D., Sierżantowicz R. (2025). Nutritional Status in a Group of Patients with Wounds Due to Diabetic Foot Disease and Chronic Venous Insufficiency. J. Clin. Med..

[10] Ceran F., Bozkurt M., Karakol P. (2025). Effectiveness of the Combined Therapy in the Treatment of Chronic Non-Healing Wounds in Patients with Autoimmune Diseases. J. Plast. Reconstr. Aesthet. Surg..

[11] Ghaffar A., Ali J. (2025). Delayed Wound Healing in Autoimmune Vasculitis: A Histopathological Study. J. Biol. Life Sci..

[12] Upputuri B., Srikantam A., Mamidi R.S. (2020). Associated with Non-Healing of Plantar Ulcers in Leprosy Patients. PLoS Negl. Trop. Dis..

[13] Hofer S.O.P., Shrayer D., Reichner J.S., Hoekstra H.J., Wanebo H.J. (1998). Wound-Induced Tumor Progression: A Probable Role in Recurrence After Tumor Resection. Arch. Surg..

[14] Padula W.V., Delarmente B.A. (2019). The National Cost of Hospital-Acquired Pressure Injuries in the United States. Int. Wound J..

[15] Bennett G., Dealey C., Posnett J. (2004). The Cost of Pressure Ulcers in the UK. Age Ageing.

[16] Ahn H., Stechmiller J., Fillingim R., Lyon D., Garvan C. (2015). Bodily Pain Intensity in Nursing Home Residents with Pressure Ulcers: Analysis of National Minimum Data Set 3.0. Res. Nurs. Health.

[17] Jackson D., Durrant L., Bishop E., Walthall H., Betteridge R., Gardner S., Coulton W., Hutchinson M., Neville S., Davidson P.M. (2017). Pain Associated with Pressure Injury: A Qualitative Study of Community-Based, Home-Dwelling Individuals. J. Adv. Nurs..

[18] Armstrong D.G., Tan T.-W., Boulton A.J.M., Bus S.A. (2023). Diabetic Foot Ulcers: A Review. JAMA.

[19] Chen L., Sun S., Gao Y., Ran X. (2023). Global Mortality of Diabetic Foot Ulcer: A Systematic Review and Meta-Analysis of Observational Studies. Diabetes Obes. Metab..

[20] Gould L., Abadir P., Brem H., Carter M., Conner-Kerr T., Davidson J., DiPietro L., Falanga V., Fife C., Gardner S. (2015). Chronic Wound Repair and Healing in Older Adults: Current Status and Future Research. Wound Repair Regen..

[21] Agrawal R., Hu A., Bollag W.B. (2023). The Skin and Inflamm-Aging. Biology.

[22] Jiao Q., Zhi L., You B., Wang G., Wu N., Jia Y. (2024). Skin Homeostasis: Mechanism and Influencing Factors. J. Cosmet. Dermatol..

[23] Rodrigues M., Kosaric N., Bonham C.A., Gurtner G.C. (2019). Wound Healing: A Cellular Perspective. Physiol. Rev..

[24] Tottoli E.M., Dorati R., Genta I., Chiesa E., Pisani S., Conti B. (2020). Skin Wound Healing Process and New Emerging Technologies for Skin Wound Care and Regeneration. Pharmaceutics.

[25] Singer A.J., Clark R.A. (1999). Cutaneous Wound Healing. N. Engl. J. Med..

[26] Xue M., Zhao R., Lin H., Jackson C. (2018). Delivery Systems of Current Biologicals for the Treatment of Chronic Cutaneous Wounds and Severe Burns. Adv. Drug Deliv. Rev..

[27] Minutti C.M., Knipper J.A., Allen J.E., Zaiss D.M.W. (2017). Tissue-Specific Contribution of Macrophages to Wound Healing. Semin. Cell Dev. Biol..

[28] Heras K.L., Igartua M., Santos-Vizcaino E., Hernandez R.M. (2020). Chronic Wounds: Current Status, Available Strategies and Emerging Therapeutic Solutions. J. Control. Release.

[29] Rousselle P., Braye F., Dayan G. (2019). Re-Epithelialization of Adult Skin Wounds: Cellular Mechanisms and Therapeutic Strategies. Adv. Drug Deliv. Rev..

[30] Schnittert J., Bansal R., Storm G., Prakash J. (2018). Integrins in Wound Healing, Fibrosis and Tumor Stroma: High Potential Targets for Therapeutics and Drug Delivery. Adv. Drug Deliv. Rev..

[31] Bonnans C., Chou J., Werb Z. (2014). Remodelling the Extracellular Matrix in Development and Disease. Nat. Rev. Mol. Cell Biol..

[32] Franklin R.A. (2021). Fibroblasts and Macrophages: Collaborators in Tissue Homeostasis. Immunol. Rev..

[33] Gurtner G.C., Werner S., Barrandon Y., Longaker M.T. (2008). Wound Repair and Regeneration. Nature.

[34] Stappenbeck T.S., Miyoshi H. (2009). The Role of Stromal Stem Cells in Tissue Regeneration and Wound Repair. Science.

[35] Guo S., DiPietro L.A. (2010). Factors Affecting Wound Healing. J. Dent. Res..

[36] Wild T., Rahbarnia A., Kellner M., Sobotka L., Eberlein T. (2010). Basics in Nutrition and Wound Healing. Nutrition.

[37] Berndt M.C., Metharom P., Andrews R.K. (2014). Primary Haemostasis: Newer Insights. Haemophilia.

[38] Smyth S.S., Mcever R.P., Weyrich A.S., Morrell C.N., Hoffman M.R., Arepally G.M., French P.A., Dauerman H.L., Becker R.C. (2009). Platelet Functions beyond Hemostasis. J. Thromb. Haemost..

[39] Özbek S., Balasubramanian P.G., Chiquet-Ehrismann R., Tucker R.P., Adams J.C. (2010). The Evolution of Extracellular Matrix. Mol. Biol. Cell.

[40] Hynes R.O. (2009). The Extracellular Matrix: Not Just Pretty Fibrils. Science.

[41] Masson-Meyers D.S., Andrade T.A.M., Caetano G.F., Guimaraes F.R., Leite M.N., Leite S.N., Frade M.A.C. (2020). Experimental Models and Methods for Cutaneous Wound Healing Assessment. Int. J. Exp. Pathol..

[42] Diller R.B., Tabor A.J. (2022). The Role of the Extracellular Matrix (ECM) in Wound Healing: A Review. Biomimetics.

[43] Miron R.J., Fujioka-Kobayashi M., Sculean A., Zhang Y. (2023). Optimization of Platelet-rich Fibrin. Periodontol..

[44] Young A., McNaught C.-E. (2011). The Physiology of Wound Healing. Surg. Oxf..

[45] Hawiger J. (1987). Formation and Regulation of Platelet and Fibrin Hemostatic Plug. Hum. Pathol..

[46] Garraud O., Hozzein W.N., Badr G. (2017). Wound Healing: Time to Look for Intelligent, ‘Natural’ Immunological Approaches?. BMC Immunol..

[47] Gefen A. (2019). How Medical Engineering Has Changed Our Understanding of Chronic Wounds and Future Prospects. Med. Eng. Phys..

[48] Lacy P. (2006). Mechanisms of Degranulation in Neutrophils. Allergy Asthma Clin. Immunol..

[49] Raziyeva K., Kim Y., Zharkinbekov Z., Kassymbek K., Jimi S., Saparov A. (2021). Immunology of Acute and Chronic Wound Healing. Biomolecules.

[50] Ferrante C.J., Leibovich S.J. (2012). Regulation of Macrophage Polarization and Wound Healing. Adv. Wound Care.

[51] Zheng H., Cheng X., Jin L., Shan S., Yang J., Zhou J. (2023). Recent Advances in Strategies to Target the Behavior of Macrophages in Wound Healing. Biomed. Pharmacother..

[52] Zaiss D.M., Minutti C.M., Knipper J.A. (2019). Immune- and Non-immune-mediated Roles of Regulatory T-cells during Wound Healing. Immunology.

[53] Hofmann U., Frantz S. (2015). Role of Lymphocytes in Myocardial Injury, Healing, and Remodeling After Myocardial Infarction. Circ. Res..

[54] Borg N., Alter C., Görldt N., Jacoby C., Ding Z., Steckel B., Quast C., Bönner F., Friebe D., Temme S. (2017). CD73 on T Cells Orchestrates Cardiac Wound Healing After Myocardial Infarction by Purinergic Metabolic Reprogramming. Circulation.

[55] Brubaker A.L., Schneider D.F., Kovacs E.J. (2011). Neutrophils and Natural Killer T Cells as Negative Regulators of Wound Healing. Expert Rev. Dermatol..

[56] Schneider D.F., Palmer J.L., Tulley J.M., Speicher J.T., Kovacs E.J., Gamelli R.L., Faunce D.E. (2011). A Novel Role for NKT Cells in Cutaneous Wound Repair. J. Surg. Res..

[57] Davis P.A., Corless D.J., Aspinall R., Wastell C. (2002). Effect of CD4+ and CD8+ Cell Depletion on Wound Healing. Br. J. Surg..

[58] McLoughlin R.M., Solinga R.M., Rich J., Zaleski K.J., Cocchiaro J.L., Risley A., Tzianabos A.O., Lee J.C. (2006). CD4+ T Cells and CXC Chemokines Modulate the Pathogenesis of Staphylococcus Aureus Wound Infections. Proc. Natl. Acad. Sci. USA.

[59] Pfisterer K., Shaw L.E., Symmank D., Weninger W. (2021). The Extracellular Matrix in Skin Inflammation and Infection. Front. Cell Dev. Biol..

[60] Beck L.S., Deguzman L., Lee W.P., Xu Y., McFatridge L.A., Amento E.P. (1991). TGF-β1 Accelerates Wound Healing: Reversal of Steroid-Impaired Healing in Rats and Rabbits. Growth Factors.

[61] Assoian R.K., Fleurdelys E.B., Stevenson H.C., Miller P.J., Madtes D.K., Raines E.W., Ross R., Sporn M.B. (1987). Expression and Secretion of Type Beta Transforming Growth Factor by Activated Human Macrophages. Proc. Natl. Acad. Sci. USA.

[62] O’Kane S., Ferguson M.W. (1997). Transforming Growth Factor βs and Wound Healing. Int. J. Biochem. Cell Biol..

[63] Shah M., Revis D., Herrick S., Baillie R., Thorgeirson S., Ferguson M., Roberts A. (1999). Role of Elevated Plasma Transforming Growth Factor-β1 Levels in Wound Healing. Am. J. Pathol..

[64] Sorokin L. (2010). The Impact of the Extracellular Matrix on Inflammation. Nat. Rev. Immunol..

[65] Si Y.-L., Zhao Y.-L., Hao H.-J., Fu X.-B., Han W.-D. (2011). MSCs: Biological Characteristics, Clinical Applications and Their Outstanding Concerns. Ageing Res. Rev..

[66] Prockop D.J., Youn Oh J. (2012). Mesenchymal Stem/Stromal Cells (MSCs): Role as Guardians of Inflammation. Mol. Ther..

[67] Bielefeld K.A., Amini-Nik S., Alman B.A. (2013). Cutaneous Wound Healing: Recruiting Developmental Pathways for Regeneration. Cell. Mol. Life Sci..

[68] Javerzat S., Auguste P., Bikfalvi A. (2002). The Role of Fibroblast Growth Factors in Vascular Development. Trends Mol. Med..

[69] Ben Amar M., Wu M. (2014). Re-Epithelialization: Advancing Epithelium Frontier during Wound Healing. J. R. Soc. Interface.

[70] Pastar I., Stojadinovic O., Yin N.C., Ramirez H., Nusbaum A.G., Sawaya A., Patel S.B., Khalid L., Isseroff R.R., Tomic-Canic M. (2014). Epithelialization in Wound Healing: A Comprehensive Review. Adv. Wound Care.

[71] El Ghalbzouri A., Hensbergen P., Gibbs S., Kempenaar J., van der Schors R., Ponec M. (2004). Facilitate Re-Epithelialization in Wounded Human Skin Equivalents. Lab. Investig..

[72] Garlick J., Parks W., Welgus H., Taichman L. (1996). Re-Epithelialization of Human Oral Keratinocytes In Vitro. J. Dent. Res..

[73] Garlick J.A., Taichman L.B. (1994). Effect of TGF-β1 on Re-Epithelialization of Human Keratinocytes In Vitro: An Organotypic Model. J. Investig. Dermatol..

[74] Gailit J., Clark R.A.F., Welch M.P. (1994). TGF-β1 Stimulates Expression of Keratinocyte Integrins During Re-Epithelialization of Cutaneous Wounds. J. Investig. Dermatol..

[75] Meng F., Cheng X., Yang L., Hou N., Yang X., Meng A. (2008). Accelerated Re-Epithelialization in Dpr2-Deficient Mice Is Associated with Enhanced Response to TGFβ Signaling. J. Cell Sci..

[76] Su D., Tsai H.-I., Xu Z., Yan F., Wu Y., Xiao Y., Liu X., Wu Y., Parvanian S., Zhu W. (2020). Exosomal PD-L1 Functions as an Immunosuppressant to Promote Wound Healing. J. Extracell. Vesicles.

[77] Tanzer M.L. (2006). Current Concepts of Extracellular Matrix. J. Orthop. Sci..

[78] Tsang K.Y., Cheung M.C.H., Chan D., Cheah K.S.E. (2010). The Developmental Roles of the Extracellular Matrix: Beyond Structure to Regulation. Cell Tissue Res..

[79] Werner S., Krieg T., Smola H. (2007). Keratinocyte–Fibroblast Interactions in Wound Healing. J. Investig. Dermatol..

[80] Ågren M.S. (1999). Matrix Metalloproteinases (MMPs) Are Required for Re-Epithelialization of Cutaneous Wounds. Arch. Dermatol. Res..

[81] Karamanos N.K., Theocharis A.D., Piperigkou Z., Manou D., Passi A., Skandalis S.S., Vynios D.H., Orian-Rousseau V., Ricard-Blum S., Schmelzer C.E. (2021). A Guide to the Composition and Functions of the Extracellular Matrix. FEBS J..

[82] Rousselle P., Montmasson M., Garnier C. (2019). Extracellular Matrix Contribution to Skin Wound Re-Epithelialization. Matrix Biol..

[83] Ricard-Blum S., Ballut L. (2011). Matricryptins Derived from Collagens and Proteoglycans. Front. Biosci..

[84] Mathew-Steiner S.S., Roy S., Sen C.K. (2021). Collagen in Wound Healing. Bioengineering.

[85] Taipale J., Keski-Oja J. (1997). Growth Factors in the Extracellular Matrix. FASEB J..

[86] Jackson-Boeters L., Wen W., Hamilton D.W. (2009). Periostin Localizes to Cells in Normal Skin, but Is Associated with the Extracellular Matrix during Wound Repair. J. Cell Commun. Signal..

[87] Peng X., Huang Y., Genin G.M. (2023). The Fibrous Character of Pericellular Matrix Mediates Cell Mechanotransduction. J. Mech. Phys. Solids.

[88] Macri L., Silverstein D., Clark R. (2007). Growth Factor Binding to the Pericellular Matrix and Its Importance in Tissue Engineering. Adv. Drug Deliv. Rev..

[89] Ehrlich H.P., Hunt T.K. (2012). Collagen Organization Critical Role in Wound Contraction. Adv. Wound Care.

[90] Baum C.L., Arpey C.J. (2006). Normal Cutaneous Wound Healing: Clinical Correlation with Cellular and Molecular Events. Dermatol. Surg..

[91] McDougall S., Dallon J., Sherratt J., Maini P. (2006). Fibroblast Migration and Collagen Deposition during Dermal Wound Healing: Mathematical Modelling and Clinical Implications. Philos. Trans. R. Soc. Math. Phys. Eng. Sci..

[92] Zeng R., Lin C., Lin Z., Chen H., Lu W., Lin C., Li H. (2018). Approaches to Cutaneous Wound Healing: Basics and Future Directions. Cell Tissue Res..

[93] Yamaguchi Y., Yoshikawa K. (2001). Cutaneous Wound Healing: An Update. J. Dermatol..

[94] Eming S.A., Martin P., Tomic-Canic M. (2014). Wound Repair and Regeneration: Mechanisms, Signaling, and Translation. Sci. Transl. Med..

[95] Bang F.S., Leeberg V., Ding M., Dreyer C.H. (2024). The Effect of VEGF Stimulation in Diabetic Foot Ulcers: A Systematic Review. Wound Repair Regen..

[96] Ozawa K., Kondo T., Hori O., Kitao Y., Stern D.M., Eisenmenger W., Ogawa S., Ohshima T. (2001). Expression of the Oxygen-Regulated Protein ORP150 Accelerates Wound Healing by Modulating Intracellular VEGF Transport. J. Clin. Investig..

[97] Mervis J.S., Phillips T.J. (2019). Pressure Ulcers: Pathophysiology, Epidemiology, Risk Factors, and Presentation. J. Am. Acad. Dermatol..

[98] Lin Z.-Q., Kondo T., Ishida Y., Takayasu T., Mukaida N. (2003). Essential Involvement of IL-6 in the Skin Wound-Healing Process as Evidenced by Delayed Wound Healing in IL-6-Deficient Mice. J. Leukoc. Biol..

[99] Ellis S., Lin E.J., Tartar D. (2018). The Basic Science of Wound Healing: Plastic and Reconstructive Surgery. Curr. Dermatol. Rep..

[100] Wang X.-J., Han G., Owens P., Siddiqui Y., Li A.G. (2006). Role of TGFβ-Mediated Inflammation in Cutaneous Wound Healing. J. Investig. Dermatol. Symp. Proc..

[101] Blakytny R., Jude E. (2006). The Molecular Biology of Chronic Wounds and Delayed Healing in Diabetes. Diabet. Med..

[102] Schultz G.S., Wysocki A. (2009). Interactions between Extracellular Matrix and Growth Factors in Wound Healing. Wound Repair Regen..

[103] Jacobsen J.N., Steffensen B., Häkkinen L., Krogfelt K.A., Larjava H.S. (2010). Skin Wound Healing in Diabetic β6 Integrin-deficient Mice. APMIS.

[104] Dabiri G., Damstetter E., Phillips T. (2016). Choosing a Wound Dressing Based on Common Wound Characteristics. Adv. Wound Care.

[105] Larouche J., Sheoran S., Maruyama K., Martino M.M. (2018). Immune Regulation of Skin Wound Healing: Mechanisms and Novel Therapeutic Targets. Adv. Wound Care.

[106] Zhao G., Usui M.L., Lippman S.I., James G.A., Stewart P.S., Fleckman P., Olerud J.E. (2013). Biofilms and Inflammation in Chronic Wounds. Adv. Wound Care.

[107] Misic A.M., Gardner S.E., Grice E.A. (2014). The Wound Microbiome: Modern Approaches to Examining the Role of Microorganisms in Impaired Chronic Wound Healing. Adv. Wound Care.

[108] Attinger C., Wolcott R. (2012). Clinically Addressing Biofilm in Chronic Wounds. Adv. Wound Care.

[109] Kirketerp-Møller K., Jensen P.Ø., Fazli M., Madsen K.G., Pedersen J., Moser C., Tolker-Nielsen T., Høiby N., Givskov M., Bjarnsholt T. (2008). Distribution, Organization, and Ecology of Bacteria in Chronic Wounds. J. Clin. Microbiol..

[110] Schreml S., Szeimies R., Prantl L., Karrer S., Landthaler M., Babilas P. (2010). Oxygen in Acute and Chronic Wound Healing. Br. J. Dermatol..

[111] Li W., Dasgeb B., Phillips T., Li Y., Chen M., Garner W., Woodley D.T. (2005). Wound-Healing Perspectives. Dermatol. Clin..

[112] Xu Z., Han S., Gu Z., Wu J. (2020). Advances and Impact of Antioxidant Hydrogel in Chronic Wound Healing. Adv. Healthc. Mater..

[113] Chin J.S., Madden L., Chew S.Y., Becker D.L. (2019). Drug Therapies and Delivery Mechanisms to Treat Perturbed Skin Wound Healing. Adv. Drug Deliv. Rev..

[114] Eming S.A., Krieg T., Davidson J.M. (2007). Inflammation in Wound Repair: Molecular and Cellular Mechanisms. J. Investig. Dermatol..

[115] Hofmann A.T., Slezak P., Neumann S., Ferguson J., Redl H., Mittermayr R. (2023). Ischemia Impaired Wound Healing Model in the Rat—Demonstrating Its Ability to Test Proangiogenic Factors. Biomedicines.

[116] Cho C.-H., Sung H.-K., Kim K.-T., Cheon H.G., Oh G.T., Hong H.J., Yoo O.-J., Koh G.Y. (2006). COMP-Angiopoietin-1 Promotes Wound Healing Through Enhanced Angiogenesis, Lymphangiogenesis, and Blood Flow in a Diabetic Mouse Model. Proc. Natl. Acad. Sci. USA.

[117] Ahn S.T., Mustoe T.A. (1990). Effects of Ischemia on Ulcer Wound Healing: A New Model in the Rabbit Ear. Ann. Plast. Surg..

[118] Kuo Y., Wang C., Wang F., Chiang Y., Wang C. (2009). Extracorporeal Shock-Wave Therapy Enhanced Wound Healing via Increasing Topical Blood Perfusion and Tissue Regeneration in a Rat Model of STZ-Induced Diabetes. Wound Repair Regen..

[119] Wu L., Mustoe T.A. (1995). Effect of Ischemia on Growth Factor Enhancement of Incisional Wound Healing. Surgery.

[120] Stadelmann W.K., Digenis A.G., Tobin G.R. (1998). Impediments to Wound Healing. Am. J. Surg..

[121] Temple W.J., Voitk A.J., Snelling C.F.T., Crispin J.S. (1975). Effect of Nutrition, Diet and Suture Material on Long Term Wound Healing. Ann. Surg..

[122] Garrow J.S. (1969). Protein Nutrition and Wound Healing. Proc. Nutr. Soc..

[123] Chow O., Barbul A. (2014). Immunonutrition: Role in Wound Healing and Tissue Regeneration. Adv. Wound Care.

[124] Attinger C., Bulan E. (2001). Débridement: The Key Initial First Step in Wound Healing. Foot Ankle Clin..

[125] Granick M., Boykin J., Gamelli R., Schultz G., Tenenhaus M. (2006). Toward a Common Language: Surgical Wound Bed Preparation and Debridement. Wound Repair Regen..

[126] Diefenbeck M., Haustedt N., Schmidt H.G. (2013). Surgical Debridement to Optimise Wound Conditions and Healing. Int. Wound J..

[127] Schiffman J., Golinko M.S., Yan A., Flattau A., Tomic-Canic M., Brem H. (2009). Operative Debridement of Pressure Ulcers. World J. Surg..

[128] Caputo W.J., Beggs D.J., DeFede J.L., Simm L., Dharma H. (2008). A Prospective Randomised Controlled Clinical Trial Comparing Hydrosurgery Debridement with Conventional Surgical Debridement in Lower Extremity Ulcers. Int. Wound J..

[129] Wilkins R.G., Unverdorben M. (2013). Wound Cleaning and Wound Healing: A Concise Review. Adv. Skin Wound Care.

[130] Han G., Ceilley R. (2017). Chronic Wound Healing: A Review of Current Management and Treatments. Adv. Ther..

[131] Hart C.E., Loewen-Rodriguez A., Lessem J. (2012). Dermagraft: Use in the Treatment of Chronic Wounds. Adv. Wound Care.

[132] Powers J.G., Higham C., Broussard K., Phillips T.J. (2016). Wound Healing and Treating Wounds: Chronic Wound Care and Management. J. Am. Acad. Dermatol..

[133] Powers J.G., Morton L.M., Phillips T.J. (2013). Dressings for Chronic Wounds. Dermatol. Ther..

[134] Nuutila K., Eriksson E. (2021). Moist Wound Healing with Commonly Available Dressings. Adv. Wound Care.

[135] Dumville J.C., Deshpande S., O’Meara S., Speak K. (2013). Foam Dressings for Healing Diabetic Foot Ulcers. Cochrane Database Syst. Rev..

[136] Ovington L.G. (2007). Advances in Wound Dressings. Clin. Dermatol..

[137] Sun G., Shen Y., Harmon J.W. (2018). Engineering Pro-Regenerative Hydrogels for Scarless Wound Healing. Adv. Healthc. Mater..

[138] Lu Y., Aimetti A.A., Langer R., Gu Z. (2016). Bioresponsive Materials. Nat. Rev. Mater..

[139] Vermonden T., Censi R., Hennink W.E. (2012). Hydrogels for Protein Delivery. Chem. Rev..

[140] Skórkowska-Telichowska K., Czemplik M., Kulma A., Szopa J. (2013). The Local Treatment and Available Dressings Designed for Chronic Wounds. J. Am. Acad. Dermatol..

[141] Gefen A., Alves P., Beeckman D., Lázaro-Martínez J.L., Lev-Tov H., Najafi B., Swanson T., Woo K. (2023). Mechanical and Contact Characteristics of Foam Materials Within Wound Dressings: Theoretical and Practical Considerations in Treatment. Int. Wound J..

[142] Gefen A., Alves P., Beeckman D., Cullen B., Lázaro-Martínez J.L., Lev-Tov H., Santamaria N., Swanson T., Woo K., Söderström B. (2024). Fluid Handling by Foam Wound Dressings: From Engineering Theory to Advanced Laboratory Performance Evaluations. Int. Wound J..

[143] Weller C.D., Team V., Sussman G. (2020). First-Line Interactive Wound Dressing Update: A Comprehensive Review of the Evidence. Front. Pharmacol..

[144] Jones A., Miguel L.S. (2013). Are Modern Wound Dressings a Clinical and Cost-Effective Alternative to the Use of Gauze?. J. Wound Care.

[145] Queen D., Orsted H., Sanada H., Sussman G. (2004). A Dressing History. Int. Wound J..

[146] Yusof N.L.B.M., Wee A., Lim L.Y., Khor E. (2003). Flexible Chitin Films as Potential Wound-Dressing Materials: Wound Model Studies. J. Biomed. Mater. Res. A.

[147] Raus R.A., Nawawi W.M.F.W., Nasaruddin R.R. (2021). Alginate and Alginate Composites for Biomedical Applications. Asian J. Pharm. Sci..

[148] Tønnesen H.H., Karlsen J. (2002). Alginate in Drug Delivery Systems. Drug Dev. Ind. Pharm..

[149] Ulijn R.V., Bibi N., Jayawarna V., Thornton P.D., Todd S.J., Mart R.J., Smith A.M., Gough J.E. (2007). Bioresponsive Hydrogels. Mater. Today.

[150] Correa S., Grosskopf A.K., Hernandez H.L., Chan D., Yu A.C., Stapleton L.M., Appel E.A. (2021). Translational Applications of Hydrogels. Chem. Rev..

[151] Mourya V., Inamdar N.N. (2008). Chitosan-Modifications and Applications: Opportunities Galore. React. Funct. Polym..

[152] Lu B., Han X., Zou D., Luo X., Liu L., Wang J., Maitz M.F., Yang P., Huang N., Zhao A. (2022). Catechol-Chitosan/Polyacrylamide Hydrogel Wound Dressing for Regulating Local Inflammation. Mater. Today Bio.

[153] Kang Y., Xu L., Dong J., Yuan X., Ye J., Fan Y., Liu B., Xie J., Ji X. (2024). Programmed Microalgae-Gel Promotes Chronic Wound Healing in Diabetes. Nat. Commun..

[154] Pranantyo D., Yeo C.K., Wu Y., Fan C., Xu X., Yip Y.S., Vos M.I.G., Mahadevegowda S.H., Lim P.L.K., Yang L. (2024). Hydrogel Dressings with Intrinsic Antibiofilm and Antioxidative Dual Functionalities Accelerate Infected Diabetic Wound Healing. Nat. Commun..

[155] Xin P., Han S., Huang J., Zhou C., Zhang J., You X., Wu J. (2023). Natural Okra-Based Hydrogel for Chronic Diabetic Wound Healing. Chin. Chem. Lett..

[156] Lu C., Sun Q., Li Z., Wei Y., Yu J., Li S., Wang Y., Li K., Tang C., Cao H. (2025). Injectable Glycyrrhizinate-Pectin Hydrogel Wound Dressing Based on Natural Ingredients. Carbohydr. Polym..

[157] Lee S.M., Park I.K., Kim Y.S., Kim H.J., Moon H., Mueller S., Jeong Y.-I. (2016). Physical, Morphological, and Wound Healing Properties of a Polyurethane Foam-Film Dressing. Biomater. Res..

[158] Liu X., Niu Y., Chen K.C., Chen S. (2017). Rapid Hemostatic and Mild Polyurethane-Urea Foam Wound Dressing for Promoting Wound Healing. Mater. Sci. Eng. C.

[159] Tran P.L., Hamood A.N., de Souza A., Schultz G., Liesenfeld B., Mehta D., Reid T.W. (2015). A Study on the Ability of Quaternary Ammonium Groups Attached to a Polyurethane Foam Wound Dressing to Inhibit Bacterial Attachment and Biofilm Formation. Wound Repair Regen..

[160] Lundin J.G., McGann C.L., Daniels G.C., Streifel B.C., Wynne J.H. (2017). Hemostatic Kaolin-Polyurethane Foam Composites for Multifunctional Wound Dressing Applications. Mater. Sci. Eng. C.

[161] Santamaria N., Gerdtz M., Sage S., McCann J., Freeman A., Vassiliou T., De Vincentis S., Ng A.W., Manias E., Liu W. (2015). A Randomised Controlled Trial of the Effectiveness of Soft Silicone Multi-Layered Foam Dressings in the Prevention of Sacral and Heel Pressure Ulcers in Trauma and Critically Ill Patients: The Border Trial. Int. Wound J..

[162] Patil P., Russo K.A., McCune J.T., Pollins A.C., Cottam M.A., Dollinger B.R., DeJulius C.R., Gupta M.K., D’aRcy R., Colazo J.M. (2022). Reactive Oxygen Species–Degradable Polythioketal Urethane Foam Dressings to Promote Porcine Skin Wound Repair. Sci. Transl. Med..

[163] Namuiriyachote N., Lipipun V., Althhatuattananglzul Y., Charoonrut P., Ritthidej G.C. (2019). Development of Polyurethane Foam Dressing Containing Silver and Asiaticoside for Healing of Dermal Wound. Asian J. Pharm. Sci..

[164] Namviriyachote N., Muangman P., Chinaroonchai K., Chuntrasakul C., Ritthidej G.C. (2020). Polyurethane-Biomacromolecule Combined Foam Dressing Containing Asiaticoside: Fabrication, Characterization and Clinical Efficacy for Traumatic Dermal Wound Treatment. Int. J. Biol. Macromol..

[165] Thomas S. (2008). Hydrocolloid Dressings in the Management of Acute Wounds: A Review of the Literature. Int. Wound J..

[166] Lee O.J., Kim J.-H., Moon B.M., Chao J.R., Yoon J., Ju H.W., Lee J.M., Park H.J., Kim D.W., Kim S.J. (2016). Fabrication and Characterization of Hydrocolloid Dressing with Silk Fibroin Nanoparticles for Wound Healing. Tissue Eng. Regen. Med..

[167] Thu H.-E., Zulfakar M.H., Ng S.-F. (2012). Alginate Based Bilayer Hydrocolloid Films as Potential Slow-Release Modern Wound Dressing. Int. J. Pharm..

[168] Chin C.-Y., Ng P.-Y., Ng S.-F. (2019). Moringa Oleifera Standardised Aqueous Leaf Extract-Loaded Hydrocolloid Film Dressing: In Vivo Dermal Safety and Wound Healing Evaluation in STZ/HFD Diabetic Rat Model. Drug Deliv. Transl. Res..

[169] Savencu I., Iurian S., Porfire A., Bogdan C., Tomuță I. (2021). Review of Advances in Polymeric Wound Dressing Films. React. Funct. Polym..

[170] Zhao Y., Huang L., Lin G., Tong M., Xie Y., Pan H., Shangguan J., Yao Q., Xu S., Xu H. (2022). Skin-Adaptive Film Dressing with Smart-Release of Growth Factors Accelerated Diabetic Wound Healing. Int. J. Biol. Macromol..

[171] Ezzelarab M.H., Nouh O., Ahmed A.N., Anany M.G., El Rachidi N.G., Salem A.S. (2019). A Randomized Control Trial Comparing Transparent Film Dressings and Conventional Occlusive Dressings for Elective Surgical Procedures. Open Access Maced. J. Med. Sci..

[172] Li C., Chang F., Gao F., Wang Y., Sun Z., Zhao L., Yang Y., Wang H., Dong L., Zheng X. (2024). Chitosan-Based Composite Film Dressings with Efficient Self-Diagnosis and Synergistically Inflammation Resolution for Accelerating Diabetic Wound Healing. Appl. Surf. Sci..

[173] Altiok D., Altiok E., Tihminlioglu F. (2010). Physical, Antibacterial and Antioxidant Properties of Chitosan Films Incorporated with Thyme Oil for Potential Wound Healing Applications. J. Mater. Sci. Mater. Med..

[174] Ranjbar R., Yousefi A. (2018). Effects of Aloe Vera and Chitosan Nanoparticle Thin-Film Membranes on Wound Healing in Full Thickness Infected Wounds with Methicillin Resistant *Staphylococcus aureus*. Bull. Emerg. Trauma.

[175] Gombotz W.R., Wee S.F. (2012). Protein Release from Alginate Matrices. Adv. Drug Deliv. Rev..

[176] Rajan D.K., Zhang L., Li H., Li J., Di X., Zhang S. (2024). Purification and Characterization of Alginate Extracted from Sargassum Hemiphyllum and Its Antioxidant and Wound Healing Efficacy. Food Biosci..

[177] Theocharidis G., Rahmani S., Lee S., Li Z., Lobao A., Kounas K., Katopodi X.-L., Wang P., Moon S., Vlachos I.S. (2022). Murine Macrophages or Their Secretome Delivered in Alginate Dressings Enhance Impaired Wound Healing in Diabetic Mice. Biomaterials.

[178] Cheng J., Wang H., Gao J., Liu X., Li M., Wu D., Liu J., Wang X., Wang Z., Tang P. (2023). First-Aid Hydrogel Wound Dressing with Reliable Hemostatic and Antibacterial Capability for Traumatic Injuries. Adv. Healthc. Mater..

[179] Min K., Tae G. (2023). Cellular Infiltration in an Injectable Sulfated Cellulose Nanocrystal Hydrogel and Efficient Angiogenesis by VEGF Loading. Biomater. Res..

[180] Yang X., Mo W., Shi Y., Fang X., Xu Y., He X., Xu Y. (2023). Fumaria Officinalis-Loaded Chitosan Nanoparticles Dispersed in an Alginate Hydrogel Promote Diabetic Wounds Healing by Upregulating VEGF, TGF-β, and b-FGF Genes: A Preclinical Investigation. Heliyon.

[181] Shao Z., Yin T., Jiang J., He Y., Xiang T., Zhou S. (2023). Wound Microenvironment Self-Adaptive Hydrogel with Efficient Angiogenesis for Promoting Diabetic Wound Healing. Bioact. Mater..

[182] Jiang T., Li Q., Qiu J., Chen J., Du S., Xu X., Wu Z., Yang X., Chen Z., Chen T. (2022). Nanobiotechnology: Applications in Chronic Wound Healing. Int. J. Nanomed..

[183] Rajendran N.K., Kumar S.S.D., Houreld N.N., Abrahamse H. (2018). A Review on Nanoparticle Based Treatment for Wound Healing. J. Drug Deliv. Sci. Technol..

[184] Wu J., Luo Y., Deng D., Su S., Li S., Xiang L., Hu Y., Wang P., Meng X. (2019). Coptisine from Coptis Chinensis Exerts Diverse Beneficial Properties: A Concise Review. J. Cell. Mol. Med..

[185] Jang M.H., Kim H.Y., Kang K.S., Yokozawa T., Park J.H. (2009). Hydroxyl Radical Scavenging Activities of Isoquinoline Alkaloids Isolated from Coptis Chinensis. Arch. Pharm. Res..

[186] Zhou R., Xiang C., Cao G., Xu H., Zhang Y., Yang H., Zhang J. (2021). Berberine Accelerated Wound Healing by Restoring TrxR1/JNK in Diabetes. Clin. Sci..

[187] Samadian H., Zamiri S., Ehterami A., Farzamfar S., Vaez A., Khastar H., Alam M., Ai A., Derakhshankhah H., Allahyari Z. (2020). Electrospun Cellulose Acetate/Gelatin Nanofibrous Wound Dressing Containing Berberine for Diabetic Foot Ulcer Healing: In Vitro and in Vivo Studies. Sci. Rep..

[188] Hu H., Luo F., Zhang Q., Xu M., Chen X., Liu Z., Xu H., Wang L., Ye F., Zhang K. (2022). Berberine Coated Biocomposite Hemostatic Film Based Alginate as Absorbable Biomaterial for Wound Healing. Int. J. Biol. Macromol..

[189] Guimarães I., Baptista-Silva S., Pintado M., Oliveira A.L. (2021). Polyphenols: A Promising Avenue in Therapeutic Solutions for Wound Care. Appl. Sci..

[190] Utpal B.K., Sutradhar B., Zehravi M., Sweilam S.H., Panigrahy U.P., Urs D., Fatima A.F., Nallasivan P.K., Chhabra G.S., Sayeed M. (2025). Polyphenols in Wound Healing: Unlocking Prospects with Clinical Applications. Naunyn. Schmiedebergs Arch. Pharmacol..

[191] Johnson J.B., Broszczak A.D., Mani J.S., Anesi J., Naiker M. (2022). A Cut above the Rest: Oxidative Stress in Chronic Wounds and the Potential Role of Polyphenols as Therapeutics. J. Pharm. Pharmacol..

[192] Zhao H., Lou Z., Chen Y., Cheng J., Wu Y., Li B., He P., Tu Y., Liu J. (2023). Tea Polyphenols (TPP) as a Promising Wound Healing Agent: TPP Exerts Multiple and Distinct Mechanisms at Different Phases of Wound Healing in a Mouse Model. Biomed. Pharmacother..

[193] Tajammal S.A., Coffey A., Tan S.P. (2025). Green Tea Polyphenols in Wound Healing: Therapeutic Mechanisms, Potential Applications and Challenges in Commercial Use for Diabetic Wound Healing. Processes.

[194] Chen G., He L., Zhang P., Zhang J., Mei X., Wang D., Zhang Y., Ren X., Chen Z. (2020). Encapsulation of Green Tea Polyphenol Nanospheres in PVA/Alginate Hydrogel for Promoting Wound Healing of Diabetic Rats by Regulating PI3K/AKT Pathway. Mater. Sci. Eng. C.

[195] Bilal K., Mehboob F., Akhtar N., Mirza I.A., Okla M.K., Dar M.J., Saleh I.A., Zomot N., Fatima H. (2024). Wound Healing, Antioxidant and Antibacterial Activities of Polyphenols of *Psidium guajava* L. Leaves. S. Afr. J. Bot..

[196] Hu B., Gao M., Boakye-Yiadom K.O., Ho W., Yu W., Xu X., Zhang X.-Q. (2021). An Intrinsically Bioactive Hydrogel with On-Demand Drug Release Behaviors for Diabetic Wound Healing. Bioact. Mater..

[197] Yu R., Yang Y., He J., Li M., Guo B. (2021). Novel Supramolecular Self-Healing Silk Fibroin-Based Hydrogel via Host–Guest Interaction as Wound Dressing to Enhance Wound Healing. Chem. Eng. J..

[198] Wei Q., Zhao Y., Wei Y., Wang Y., Jin Z., Ma G., Jiang Y., Zhang W., Hu Z. (2023). Facile Preparation of Polyphenol-Crosslinked Chitosan-Based Hydrogels for Cutaneous Wound Repair. Int. J. Biol. Macromol..

[199] Quan L., Xin Y., Zhang H., Wu X., Li X., Zhou C., Ao Q. (2025). Polyphenol Enhances the Functionality of Borate Hydrogel in Wound Repair by Regulating the Wound Microenvironment. Colloids Surf. B Biointerfaces.

[200] Cao Y.-J., Pu Z.-J., Tang Y.-P., Shen J., Chen Y.-Y., Kang A., Zhou G.-S., Duan J.-A. (2017). Advances in Bio-Active Constituents, Pharmacology and Clinical Applications of Rhubarb. Chin. Med..

[201] Wang S., Zhang Y., Shi Y., He Q., Tan Q., Peng Z., Liu Y., Li D., Li X., Ke D. (2023). Rhubarb Charcoal-Crosslinked Chitosan/Silk Fibroin Sponge Scaffold with Efficient Hemostasis, Inflammation, and Angiogenesis for Promoting Diabetic Wound Healing. Int. J. Biol. Macromol..

[202] Tan Q., He Q., Peng Z., Zeng X., Liu Y., Li D., Wang S., Wang J. (2024). Topical Rhubarb Charcoal-Crosslinked Chitosan/Silk Fibroin Sponge Scaffold for the Repair of Diabetic Ulcers Improves Hepatic Lipid Deposition in Db/Db Mice via the AMPK Signalling Pathway. Lipids Health Dis..

[203] Liu C. (2015). Inhibition of Mechanical Stress-Induced Hypertrophic Scar Inflammation by Emodin. Mol. Med. Rep..

[204] Guan R., Wang X., Zhao X., Song N., Zhu J., Wang J., Wang J., Xia C., Chen Y., Zhu D. (2016). Emodin Ameliorates Bleomycin-Induced Pulmonary Fibrosis in Rats by Suppressing Epithelial-Mesenchymal Transition and Fibroblast Activation. Sci. Rep..

[205] Yang W.-T., Ke C.-Y., Wu W.-T., Tseng Y.-H., Lee R.-P. (2020). Antimicrobial and Anti-Inflammatory Potential of Angelica Dahurica and Rheum Officinale Extract Accelerates Wound Healing in Staphylococcus Aureus-Infected Wounds. Sci. Rep..

[206] Yang W.-T., Ke C.-Y., Wu W.-T., Harn H.-J., Tseng Y.-H., Lee R.-P. (2017). Effects of *Angelica dahurica* and *Rheum officinale* Extracts on Excisional Wound Healing in Rats. Evid. Based Complement Alternat. Med..

[207] Aduba D.C., Yang H. (2017). Polysaccharide Fabrication Platforms and Biocompatibility Assessment as Candidate Wound Dressing Materials. Bioengineering.

[208] Gong H., Li W., Sun J., Jia L., Guan Q., Guo Y., Wang Y. (2022). A Review on Plant Polysaccharide Based on Drug Delivery System for Construction and Application, with Emphasis on Traditional Chinese Medicine Polysaccharide. Int. J. Biol. Macromol..

[209] He X., Wang X., Fang J., Zhao Z., Huang L., Guo H., Zheng X. (2017). *Bletilla striata*: Medicinal Uses, Phytochemistry and Pharmacological Activities. J. Ethnopharmacol..

[210] Xu D., Pan Y., Chen J. (2019). Chemical Constituents, Pharmacologic Properties, and Clinical Applications of *Bletilla striata*. Front. Pharmacol..

[211] Chen H., Zeng J., Wang B., Cheng Z., Xu J., Gao W., Chen K. (2021). Structural Characterization and Antioxidant Activities of *Bletilla striata* Polysaccharide Extracted by Different Methods. Carbohydr. Polym..

[212] Zhang H.-Y., Wang K.-T., Zhang Y., Cui Y.-L., Wang Q. (2023). A Self-Healing Hydrogel Wound Dressing Based on Oxidized *Bletilla striata* Polysaccharide and Cationic Gelatin for Skin Trauma Treatment. Int. J. Biol. Macromol..

[213] Zhang Q., Qi C., Wang H., Xiao X., Zhuang Y., Gu S., Zhou Y., Wang L., Yang H., Xu W. (2019). Biocompatible and Degradable *Bletilla striata* Polysaccharide Hemostasis Sponges Constructed from Natural Medicinal Herb Bletilla Striata. Carbohydr. Polym..

[214] He X., Liu L., Gu F., Huang R., Liu L., Nian Y., Zhang Y., Song C. (2024). Exploration of the Anti-Inflammatory, Analgesic, and Wound Healing Activities of *Bletilla striata* Polysaccharide. Int. J. Biol. Macromol..

